# Glyco-engineered cell line and computational docking studies reveals enterotoxigenic *Escherichia coli* CFA/I fimbriae bind to Lewis a glycans

**DOI:** 10.1038/s41598-018-29258-0

**Published:** 2018-07-26

**Authors:** Lynda Mottram, Jining Liu, Sonali Chavan, Joshua Tobias, Ann-Mari Svennerholm, Jan Holgersson

**Affiliations:** 1 Department of Microbiology and Immunology at the Institute of Biomedicine, Sahlgrenska Academy, University of Gothenburg, Gothenburg, Sweden; 2 Department of Clinical Chemistry and Transfusion Medicine at the Institute of Biomedicine, Sahlgrenska Academy, University of Gothenburg, Gothenburg, Sweden; 3 Department of Chemistry and Molecular Biology, University of Gothenburg, Gothenburg, Sweden; 4 Institute of Specific Prophylaxis and Tropical Medicine, Medical University of Vienna, Vienna, Austria

## Abstract

We have previously reported clinical data to suggest that colonization factor I (CFA/I) fimbriae of enterotoxigenic *Escherichia coli* (ETEC) can bind to Lewis a (Le^a^), a glycan epitope ubiquitous in the small intestinal mucosa of young children (<2 years of age), and individuals with a genetic mutation of *FUT2*. To further elucidate the physiological binding properties of this interaction, we engineered Chinese Hamster Ovary (CHO-K1) cells to express Le^a^ or Le^b^ determinants on both *N*- and *O*-glycans. We used our glyco-engineered CHO-K1 cell lines to demonstrate that CfaB, the major subunit of ETEC CFA/I fimbriae, as well as four related ETEC fimbriae, bind more to our CHO-K1 cell-line expressing Le^a^, compared to cells carrying Le^b^ or the CHO-K1 wild-type glycan phenotype. Furthermore, using *in-silico* docking analysis, we predict up to three amino acids (Glu^25^, Asn^27^, Thr^29^) found in the immunoglobulin (Ig)-like groove region of CfaB of CFA/I and related fimbriae, could be important for the preferential and higher affinity binding of CFA/I fimbriae to the potentially structurally flexible Le^a^ glycan. These findings may lead to a better molecular understanding of ETEC pathogenesis, aiding in the development of vaccines and/or anti-infection therapeutics.

## Introduction

Enterotoxigenic *Escherichia coli* (ETEC) is a leading cause of severe diarrhoeal illness in young children (<5 years of age) in low and middle-income countries. It is also a major cause of traveller’s diarrhoea to ETEC endemic areas^1,2^. The bacterium has evolved to produce one or more of at least 23 distinct fimbrial (known as colonisation factors, CFs) or non-fimbrial adhesins, enabling ETEC to bind to the small intestinal epithelium before producing diarrhoeagenic enterotoxin(s)^3^. Thus, ETEC adherence factors are prerequisites for the initiation of pathogenesis, representing a critical point at which ETEC infections could be prevented^4^.

In previous clinical studies, we have demonstrated that Bangladeshi children expressing the histo-blood group antigen (HBGA) Lewis a (Le^a^, Le(a + b−) phenotype, *FUT2* non-secretor status) are more likely to have symptomatic ETEC infection compared to children expressing the HBGA Lewis b (Le^b^, Le(a−b+) phenotype, *FUT2* secretor status)^5,6^. Interestingly, we have also observed that Bangladeshi children with the Le(a+ b−) phenotype are more likely to be infected by ETEC expressing the colonisation factor antigen I (CFA/I) and the related ETEC CF family fimbriae or pili^6^. The likely explanation for this being, CFA/I could bind to Le^a^ glycolipid structures present in the small intestinal mucosal layer of very young children (<2 years of age) and individuals with *FUT2* non-secretor status^7,8^.

CFA/I was the first human specific immunogenic ETEC CF to be described. It is a representative member of the antigenically defined ETEC CF class 5 pili, which are also commonly referred to as the α clade fimbrial usher protein (FUP) family^4,9^. Together, this ETEC CF group (CFA/I, CS1, CS2, CS4, CS14, CS17, CS19 and PCF071) accounts for the largest group of human specific ETEC CF expressing strains causing diarrhoeal disease worldwide^2,4^. Like other ETEC CF family members, CFA/I is comprised of a four gene operon, encoding for a long rigid homopolymorphic shaft with >1,000 copies of a major subunit (CfaB), with one or a few copies of the tip residing minor subunit (CfaE)^4^.

With regard to ETEC CFA/I binding to host cells, the minor subunit CfaE binds to the surface of erythrocytes^4,10^. The major subunit CfaB has been shown to bind to glycosphingolipids and human small intestinal glycolipid structures, such as those expressing Le^a^ or asialo-GM1^7^. It has also been shown that specific monoclonal antibodies raised against CfaB inhibits ETEC CFA/I binding to cultured intestinal epithelial cells^11–13^. Moreover, an antibody that reacts strongly with the first 25 amino acids of the N-terminal fragment of CfaB has been shown to inhibit ETEC CFA/I bacterial adhesion to human jejunal enterocytes^14,15^. In contrary, it has been reported by others that CfaE of ETEC CFA/I binds to intestinal tissue and asialo-GM1 glycans that are expressed on erythrocytes and cultured intestinal epithelial cells^10,16^.

X-ray structural analysis has revealed CfaE and CfaB to have similar barrel like structures, with CfaE containing two and CfaB possessing one exposed hydrophobic immunoglobulin (Ig)-like fold(s), that structurally interact and complement each other^9^. Interestingly, a 12-amino acid stretch of the CfaB Ig-like fold (V^24^EKNITVTASVD^35^) that is located in the N terminal fragment of CfaB, shares structural similarities with all ETEC CF major subunits of the type 5 pili family. This 12 amino acid stretch of CfaB also shared structural similarities with class 1 pili from bacteria that can cause urinary and respiratory infections by binding to host glycolipids containing HBGAs^9,17^.

The aim of the present study was to create glycan defined Chinese hamster ovary (CHO-K1) cell line models of the human small intestinal mucosa, and to study the binding capabilities of ETEC CFA/I and the related CFs to Lewis Le^a^ and Le^b^ antigens expressed on the cell surface. We also perform computational molecular docking analysis to help understand why CFA/I binds to Le^a^ but not Le^b^ expressing glycans, as well as potentially identify novel CFA/I Le^a^ glycan binding sites.

## Results

### Glyco-engineered CHO-K1 cells were produced expressing either Le^a^ or Le^b^

ETEC colonises the epithelial surface of the small intestinal mucosa, where intestinal villi and crypts express abundant Le^a^ and/or Le^b^ glycans^4,18,19^. To create defined HBGA Le^a^ and Le^b^ glycan models of the human small intestinal mucosa, CHO-K1 cells expressing the P-selectin glycoprotein ligand-1/immunoglobulin fusion protein (PSGL-1/mIgG2b; CHO-CP55 cells), were co-transfected with plasmids encoding: the extended core 1 glycan (GlcNAcβ3Galβ3GalNAcα) enzyme B3GNT3, the type 1 chain glycan (Galβ3GlcNAc) encoding enzyme B3GALT5, and the Lewis gene-encoding enzyme FUT3 alone (generating Le^a^ cells; CHO-PSGL-Le^a^-a1) or together with the H gene-encoding FUT1 (generating Le^b^; CHO-PSGL-Le^b^-b1) (see Supplementary Figure S1 and Table S1 online, and Materials and Methods for further details)^20–24^.

**Figure 1:**
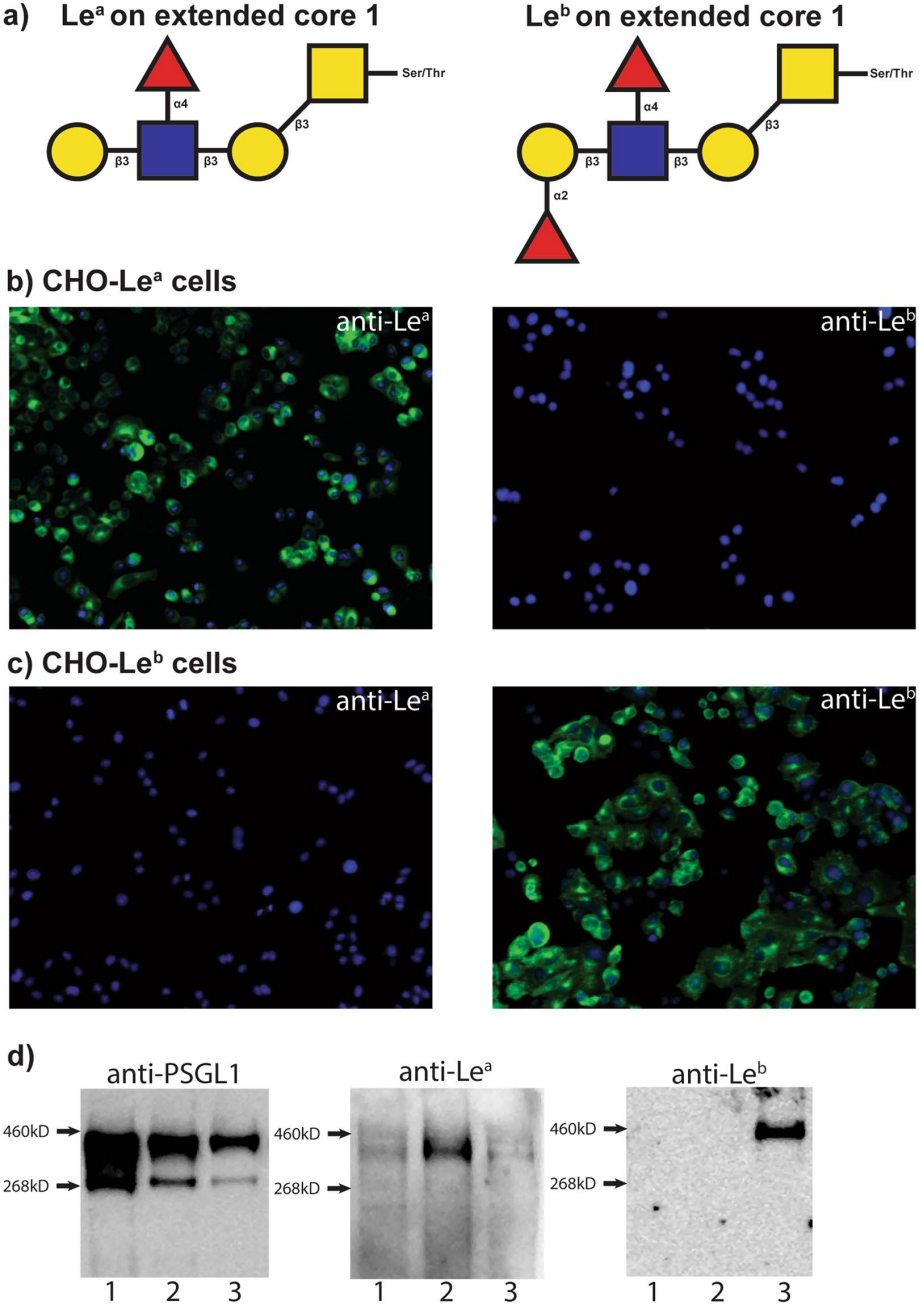
Glyco-engineered CHO-Le^a^ and CHO-Le^b^ cell lines express Lewis antigens. (**a**) SNFG (Symbol Nomenclature for Glycans) diagrams of the Le^a^ and Le^b^ expressing glycan structures that were engineered to CHO-K1 cell lines for this study. For further details please also see Supplementary Fig. S1 and Table S1 online. [(**b**,**c**)] Immunocytochemistry staining of the CHO-Le^a^ and CHO-Le^b^ cell lines. (**b**) CHO-Le^a^ cells stained with anti-Le^a^ and anti-Le^b^ antibodies. (**c**) CHO-Le^b^ cells stained with anti-Le^a^ and anti-Le^b^ antibodies. Lewis antigens are visualized with Alexa Fluor 488-conjugated antibodies (green) and host cell nuclei with DAPI (blue). Magnification x20. (**d**) SDS-PAGE and Western blot analysis of PSGL-1/mIgG2b proteins expressed in the CHO-CP55 (lane 1), CHO-Le^a^ (lane 2) and CHO-Le^b^ (lane 3) cell lines. In each lane, 1.5 μg of protein was loaded. Membranes were probed with either anti-PGSL1, anti-Le^a^ or anti Le^b^ antibodies followed by an anti-mouse IgG secondary antibody.

Immunocytochemistry with anti-Le^a^ and anti-Le^b^ antibodies showed that all DAPI stained cells of the selected and expanded clone CHO-PSGL1-Le^a^-a1 were positively stained (in green) for Le^a^, but not for Le^b^ (Fig. 1b). Similarly, the DAPI stained CHO-PSGL1-Le^b^-b4 clone was only positively stained for Le^b^ (Fig. 1c). Subsequent analysis by SDS-PAGE and Western blotting of the purified PSGL-1/mIgG2b produced by these clones, revealed CHO-PSGL1-Le^a^-a1 expressed only Le^a^, whereas the CHO-PSGL1-Le^b^-b4 clone predominantly expressed Le^b^ (Fig. 1d and Supplementary Fig S7 online). Therefore, the CHO-PSGL1-Le^a^-a1 (CHO-Le^a^), CHO-PSGL1-Le^b^-b4 (CHO-Le^b^) cell lines along with the Lewis antigen negative control cell lines CHO-CP55 and CHO-K1, were used for the ETEC CF-binding characterization experiments.

### ETEC CFA/I fimbriae binds to CHO-Le^a^ cells

**Figure 2:**
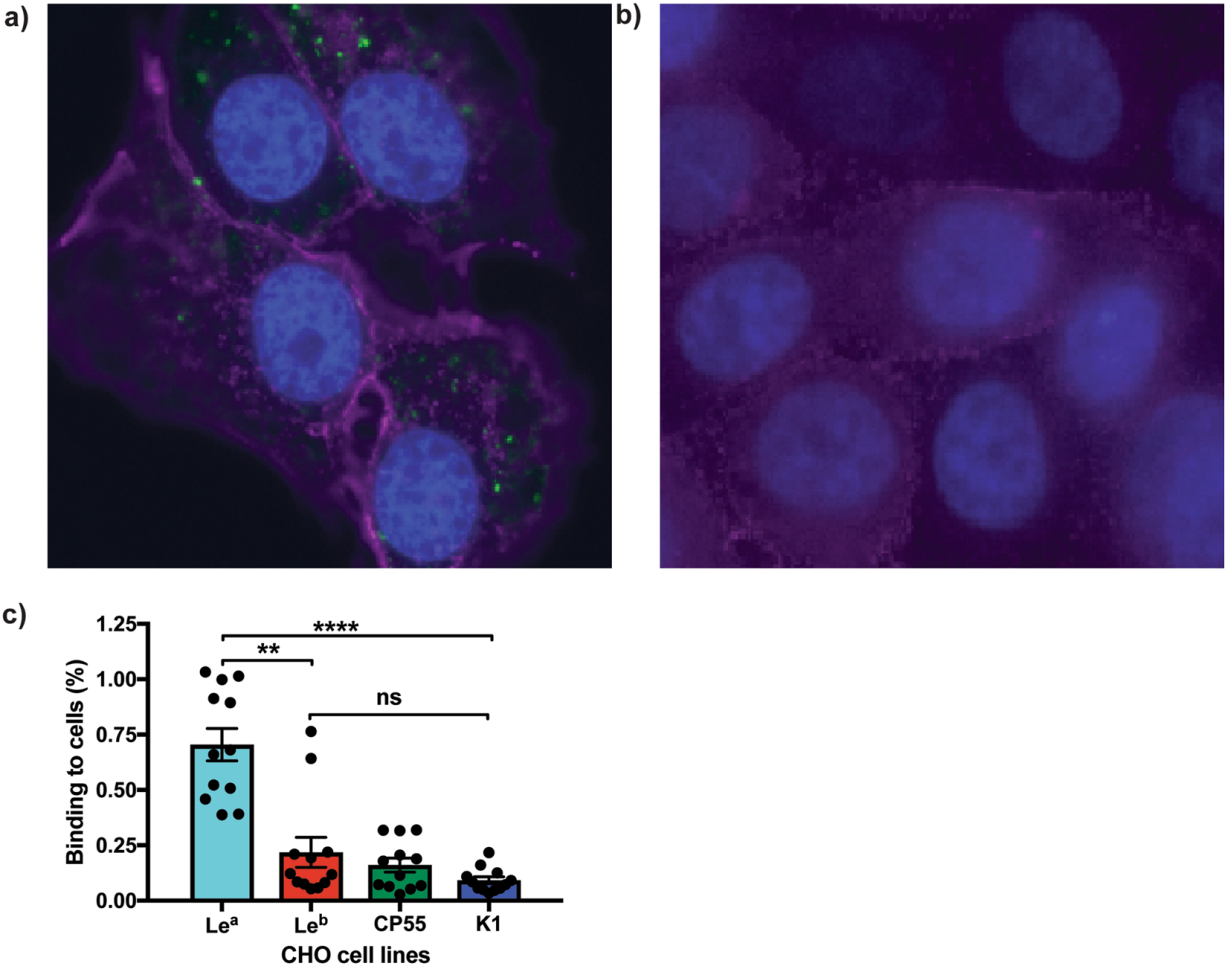
ETEC CFA/I fimbriae bind to the CHO-Le^a^ cells. Immunofluorescence staining of (**a**) CHO-Le^a^ and (**b**) CHO-Le^b^ cells infected with TOP10-CFA/I. Infected cell lines were stained with anti-CFA/I antibody (Alexa Fluor 488, green), Le^a^ antibody (Alexa Fluor 647, purple), Le^b^ antibody (Texas Red, purple) and host nuclei stain (DAPI, Blue). All images are taken at x40 magnification. (**c**) TOP10-CFA/I bacteria adhere to the CHO-Le^a^ cell line more than to CHO-Le^b^, CHO-CP55 and CHO-K1 cells. Graphs represent the percentage of TOP10-CFA/I bacteria binding to each of the cell lines, as measured by quantifiable immunofluorescence analysis. Statistical analysis was performed using ANOVA with Dunn’s multiple comparisons test. **Indicates *P* = 0.0065, ****Indicates *P* = <0.0001. Data presented as Mean and SEM of at least three independent experiments.

In previous studies, we have demonstrated that ETEC CFA/I fimbriae bind to Le^a^-5 glycolipids separated onto thin layer chromatograms^7^. To evaluate the binding specificity of ETEC CFA/I fimbriae to defined Le^a^ or Le^b^ expressing glycans, the glyco-engineered CHO-K1 cells expressing either Le^a^ (CHO-Le^a^) or Le^b^ (CHO-Le^b^) determinants on their cell surface, along with the negative glycan phenotype control cell lines (CHO-CP55 and CHO-K1) were infected with a recombinant *E. coli* Top10-CFA/I bacterial strain. Using immunocytochemistry, we observed the CFA/I strain attached more to CHO-Le^a^ (Fig. 2a) than to CHO-Le^b^ cells (Fig. 2b). Furthermore, when we measured the binding of the recombinant CFA/I strain using a quantitative immunofluorescence assay (Fig. 2c), we observed approximately three-fold, significantly higher, bacterial binding to the CHO-Le^a^ cell line than to the CHO-Le^b^ (*P* = 0.0065), CHO-CP55 and CHO-K1 cell lines (*P* = <0.0001).

### The ETEC CFA/I major subunit CfaB binds more to CHO-Le^a^ cells than the ETEC CFA/I minor subunit CfaE

**Figure 3:**
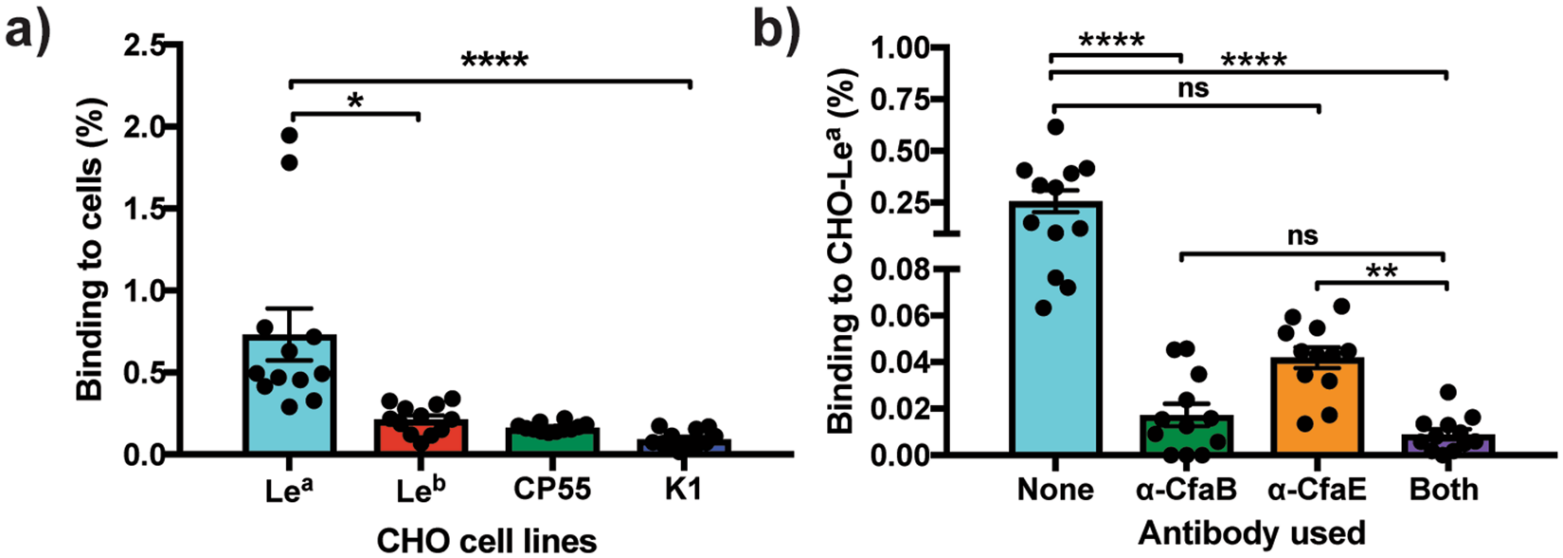
CfaB, the major subunit of ETEC CFA/I fimbriae, binds to the CHO-Le^a^ cells. (**a**) TOP10-CFA/IΔE bacteria adhere to the CHO-Le^a^ cell line more than to CHO-Le^b^, CHO-CP55 and CHO-K1 cell lines. (**b**) Inhibition of binding of TOP10-CFA/I bacteria to the CHO-Le^a^ cells, using MAbs specific for the CFA/I major subunit CfaB, the minor subunit CfaE, and a mixture of anti-CfaB and anti-CfaE. Graphs represent the percentage of bacteria binding to each of the cell lines, as measured by quantifiable immunofluorescence analysis. Statistical analysis was performed using ANOVA with Dunn’s multiple comparisons test. *Indicates *P* = 0.0376, **Indicates *P* = 0.0035, ****Indicates *P* = <0.0001. Data presented as Mean and SEM of (**a**) three independent and (**b**) two independent experiments.

It has previously been reported that CFA/I has two distinct binding activities with CfaE binding to receptors of unknown structures on the surface of erythrocytes and intestinal epithelial and cultured cells, whilst the major subunit CfaB binds to various glycolipids present on human small intestinal tissue^4,7,16^. To assess if either the major CfaB subunit or the minor CfaE subunit are responsible for the binding of CFA/I to Le^a^, we infected our glyco-engineered cell lines with a CFA/I recombinant strain without the minor subunit CfaE (Top10-CFA/IΔ*E*). Using our semi-quantitative immunofluorescence assay, we found that the CFA/IΔ*E* strain bound at a significantly higher percentage to the CHO-Le^a^ cell line compared to the CHO-Le^b^ (Fig. 3a, *P* = 0.0376), CHO-CP55 and CHO-K1 cell lines (Fig. 3a, *P* = <0.0001).

To substantiate these observations, we next performed an inhibition assessment assay using anti-CfaB and anti-CfaE antibodies, the Top10-CFA/I bacterial strain and CHO-Le^a^ cells. We found that pre-incubation of the TOP10-CFA/I strain with the anti-CfaB antibody alone, or in combination with equal amounts of the anti-CfaE antibody, significantly reduced the binding of the Top10-CFA/I strain to the CHO-Le^a^ cells (Fig. 3b, *P* = <0.0001). There was a slight but no significant decrease (*P* = <0.08) in the binding to CHO-Le^a^ cells when the Top10-CFA/I strain was pre-incubated with the anti-CfaE antibody alone, compared to no antibody incubation (Fig. 3b).

### ETEC CFA/I CF family members also bind to CHO-Le^a^ cells

**Figure 4:**
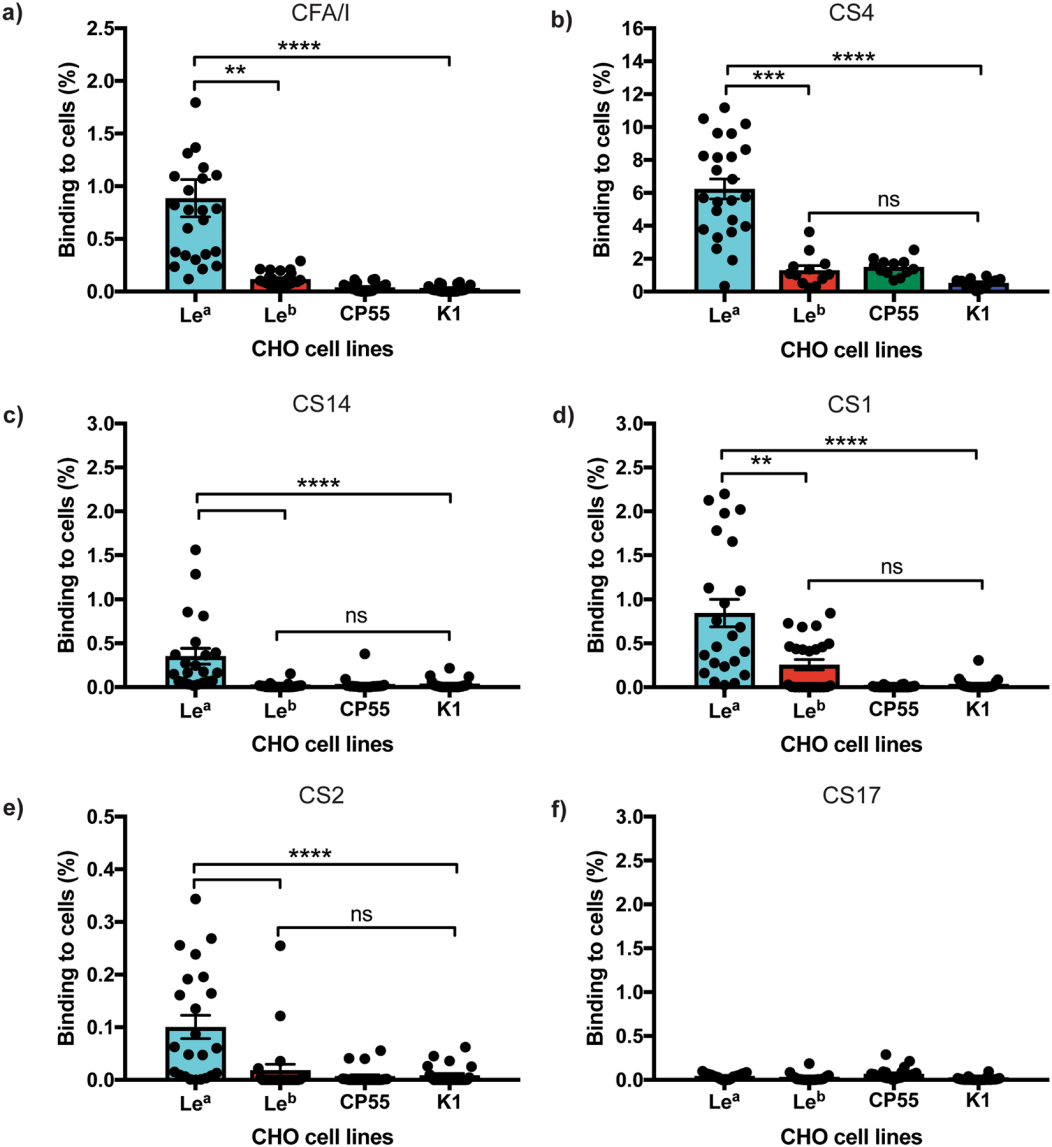
ETEC CFA/I and other type 5 pili family members bind to CHO-Le^a^ cells. (**a**) ETEC expressing CFA/I^+^ ST^+^/LT^+^ (**b**) ETEC expressing CS4^+^, ST^−^/LT^−^ (**c**) ETEC expressing CS14^+^, STh^+^/LT^−^ (**d**) ETEC expressing CS1^+^, ST^−^/LT^−^ (**e**) ETEC expressing CS2^+^, ST^−^/LT^−^, and (**f)** ETEC expressing CS17^+^, ST^−^/LT^+^ and their binding to CHO-Le^a^, CHO-Le^b^, CHO-CP55 and CHO-K1 cells. Graphs represent the percentage of bacteria binding to each of the cell lines, as measured by quantifiable immunofluorescence analysis. Statistical analysis was performed using ANOVA with Dunn’s multiple comparisons test. **Indicates *P* = <0.0040, ***Indicates *P* = 0.0002, ****Indicates *P* = <0.0001. Data represent the Mean and SEM of two independent experiments.

We have previously demonstrated that Bangladeshi children expressing the Le^a^ antigen (Le(a + b−), non-secretor phenotype) are more likely to be suffering with symptomatic ETEC infection if they have been infected by ETEC strains expressing CFA/I, CS1, CS2, CS4, CS14 and CS17 CFs^7^. To highlight the flexibility of our glycan models, we also infected them with wild-type ETEC reference strains expressing the above CFs (see materials and methods for further details of the ETEC reference stains used). We observed the wild-type ETEC strain expressing CFA/I bound at a significantly higher percentage to CHO-Le^a^ cells, compared to the CHO-Le^b^ (*P* = 0.0040, Fig. 4), CHO-CP55 and CHO-K1 cells (*P* = <0.0001, Fig. 4). Similarly, CS4 expressing ETEC bacteria attached strongly to Le^a^ but not the Le^b^ or negative control expressing CHO cells (*P* = <0.0002, Fig. 4). Moreover, the wild-type ETEC strains expressing CS1, CS2, and CS14 adhered significantly more to the CHO-Le^a^ than to the CHO-Le^b^, CHO-CP55 and CHO-K1 cells (P = <0.0040, Fig. 4). However, we could see no significant difference in the binding of the ETEC strain expressing CS17 to any of our glycan cell line models (Fig. 4).

### Molecular docking predicts the Ig-like groove of CfaB is used by ETEC CFA/I to bind to Le^a^ expressing glycans

**Figure 5:**
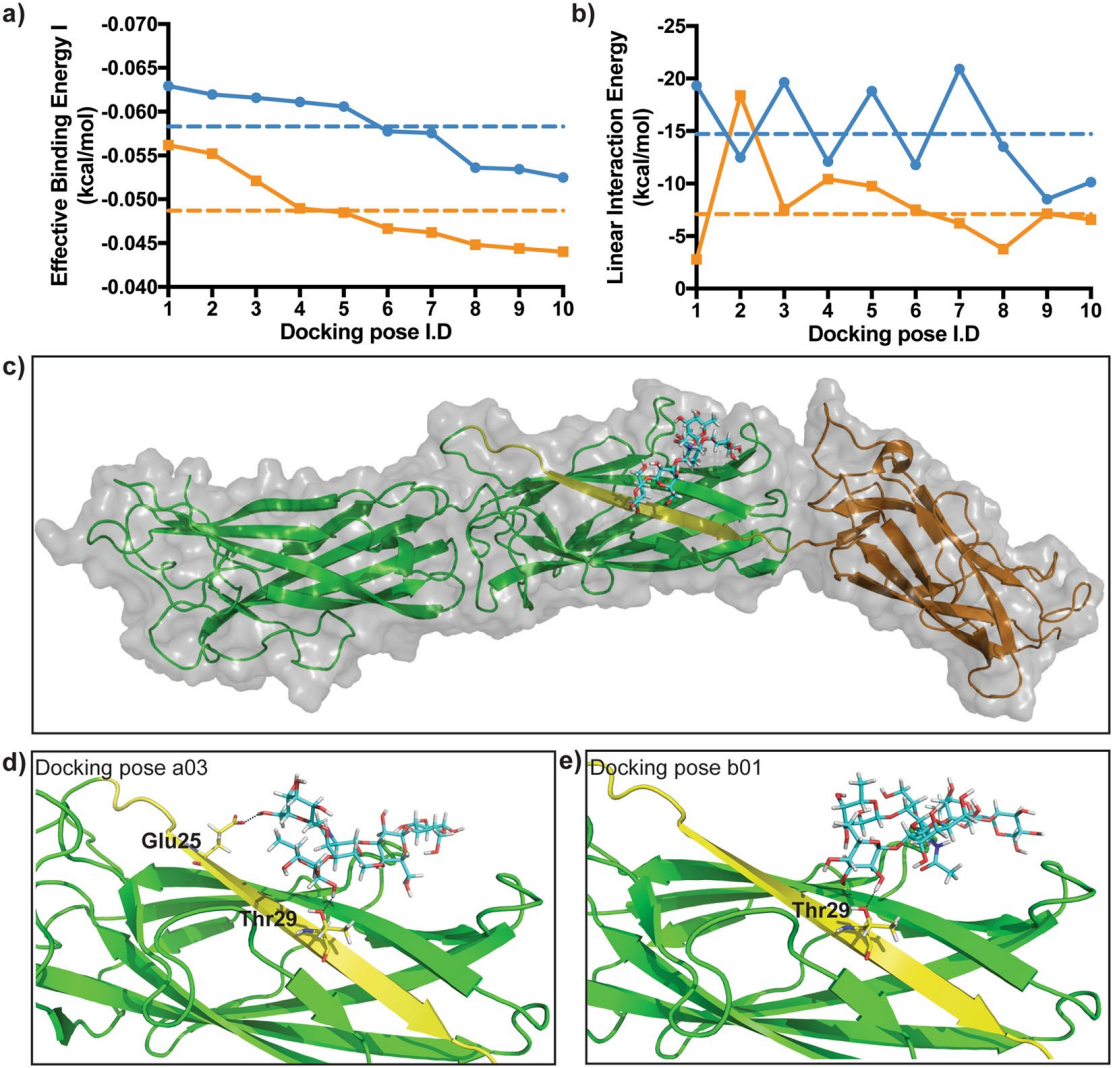
Molecular docking predicts CfaB can bind to Le^a^-5 and Le^b^-6 glycans. (**a**) Effective Binding Energy I (kcal/mol) of the ten highest ranked Le^a^-5 and Le^b^-6 poses. (**b**) Linear Interaction Energy (kcal/mol) of the ten highest ranked Le^a^-5 and Le^b^-6 poses. Blue lines are Le^a^-5 poses and orange lines are Le^b^-6 poses. Dotted lines are respective mean values for the ten highest ranked Le^a^-5 (blue) and Le^b^-6 (orange) poses analysed. (**c**) Overall surface view and cartoon representation of Le^a^-5 binding to the N-terminal Ig-like groove of the major CFA/I subunit CfaB. (**d**) Cartoon representation of docking pose a03, showing hydrogen bond interaction of residues Glu^25^ and Thr^29^ of the CfaB Ig-like groove with Le^a^-5. (**e**) Cartoon representation of docked complex b01, showing hydrogen bond interaction of Thr^29^ of the CfaB Ig-like groove with Le^b^-6. For (**c**) to e) the Le^a^-5 and Le^b^-6 ligands are stick representations (cyan) with atoms interacting with CfaB coloured in yellow and hydrogen bonds displayed as black dotted lines. For the CFA/I fimbriae, the minor subunit CfaE is the green ribbon, the major subunit CfaB is the dark yellow ribbon and the CfaB N terminal Ig-like groove is the bright yellow ribbon.

To provide an insight into why CFA/I fimbriae can bind to Le^a^ glycans, we performed molecular docking simulations using pre-existing 3D protein and ligand structures of CFA/I and the Le^a^-5 and Le^b^-6 determinants^9,25–27^. For each glycan, 32 docking simulations were generated around the N terminal Ig-like region of the CfaB major subunit of CFA/I (see Fig. 5c as an example). To predict the most likely of these docking simulations, these poses were ranked based on highest Effective Binding Energy (I and II) (see Supplementary Tables S2 and S3 online, and materials and methods for further details).

Interestingly, we noticed the ten most likely Le^a^-5 poses had relatively higher Effective Binding Energy than the ten most likely Le^b^-6 poses (Fig. 5a and Supplementary Tables S2 and S3 online). Equally, these Le^a^-5 docking poses had on average, higher Linear Interaction Energy than the Le^b^-6 docking poses, suggesting Le^a^-5 interacts more strongly with the CFA/I docking site, compared to Le^b^-6.

Next, we examined if the Ig-like groove region of CfaB could be binding to the Lewis antigen containing regions of our most likely Le^a^-5 and Le^b^-6 docking poses. From this selection, we instantly identified the docking candidate with the highest Effective Binding Energy (I and II) and Linear Interaction Energy to be the Le^a^-5 docking pose, a03 (Table 1). This selection also predicted Thr^29^ and up to two other amino acids (Glu^25^ Asn^27^) of the CfaB Ig-like groove V^24^EKNITVTASVD^35^ region could be binding to the α1,4-FucT (Le^a^) and/or the neighboring β1,3 Gal and GalNAc moieties of the Le^a^-5 glycan (Table 1, Fig. 5d, Supplementary Figs S3 and S4 online). In contrast to the most likely Le^b^-6 docking poses (b01,b05, b07 and b08), we observed that only one CfaB amino acid (Thr^29^) might bind to just the α1,2-FucT moiety (Le^b^) of the Le^b^-6 glycan (Table 1, Fig. 5e, Supplementary Figs S3 and S5 online). Furthermore, the comparatively higher ligand strain energy, the lower ligand strain energy scores and relatively high ligand solvent GB scores of Le^a^-5 compared to Le^b^-6 (Table 1), implies less steric hindrance of Le^a^-5 in the CFA/I docking site suggesting that Le^a^-5 is a more distorted structure than the Le^b^-6 glycan.

**Table 1:** *In-silico* docking analysis predicts relatively higher binding affinity of the CFA/I docking site to Le^a^-5.

Pose I.D	XP Glide Score	MM-GBSA score	Effective Binding Energy I^a^	Effective Binding Energy II^b^	Ligand solvent GB	Ligand strain energy	Linear Interaction Energy^c^
**Selected Le** ^**a**^ **-5 docking poses**							
a03	−8.898	−52.583	−0.061	−0.907	−32.956	7.368	−19.627
a04	−10.036	−52.168	−0.061	−0.899	−40.076	15.161	−12.092
a06	−10.182	−49.319	−0.058	−0.850	−37.542	18.196	−11.777
a08	−10.310	−45.768	−0.054	−0.789	−32.258	14.674	−13.509
a09	−8.005	−45.628	−0.053	−0.787	−37.112	10.394	−8.517
**Selected Le**^**b**^-**6 docking poses**							
b01	−8.302	−56.176	−0.056	−0.826	−53.394	0.935	−2.782
b02	−8.798	−55.204	−0.055	−0.812	−36.825	0.081	−18.379
b05	−8.395	−48.488	−0.048	−0.713	−38.728	11.456	−9.759
b07	−8.046	−46.198	−0.046	−0.679	−39.974	5.267	−6.224
b08	−9.352	−44.813	−0.045	−0.659	−41.044	5.922	−3.769

Candidate poses selected from the ten highest ranked Le^a^-5 and Le^b^-6 docking scores based on lowest Effective Binding Energy I and II, that predict CfaB binding near/on Lewis antigen moieties of either the Le^a^-5 or Le^b^-6 glycan. All energy score units are measured in kcal/mol.

^a^MM-GBSA score/molecular weight of glycan.

^b^MM-GBSA score/number of heavy atoms in glycan.

^c^MM-GBSA score - Ligand solvent GB.

## Discussion

The surface of the mammalian intestinal tract is covered in a rich diversity of mucosal glycans. Such glycans often express genetically defined HBGAs, with HBGA expression varying in the small and large intestine. HBGA glycan expression evolves from birth, and can contribute to intestinal homeostasis, and microbial composition. However, these genetically defined HBGAs can also act as target binding receptors for the virulence factors of microbial pathogens. Such host-pathogen interactions are thought to contribute to pathogen-host species specify, and pathogen-host tissue tropism^17–19,28^. For example, *FUT2*^−*/*−^ (i.e. HBGA Le^a^-expressing) individuals are genetically immune to certain norovirus genotypes (GI-1, G-II-3 and G-II-4) compared to *FUT2*^+*/*+^ or *FUT2*^+*/*−^ individuals^17^. Similarly *FUT2*^+/+^ pigs are more susceptible to porcine pathogenic ETEC strains expressing F18^+^ fimbriae^29^.

We have previously published clinical evidence to suggest that ETEC CFA/I uses the HBGA Le^a^ as a small intestinal host receptor, as Bangladeshi children expressing this Le^a^ antigen (caused by a *FUT2* mutation, non-secretor status) are more susceptible to disease caused by ETEC expressing CFA/I and related CF family members, than are Bangladeshi children expressing the Le^b^ antigen (functional *FUT2*, secretor status)^5–7^. Expression of HBGAs (Lewis and ABO(H)) on the surface of the human intestinal mucosa is driven by the *FUT2* and *FUT3* genes, with *FUT2* encoding an α1,2-fucosyltransferase (α1,2-FucT) and *FUT3* encoding an α1,3/4-fucosyltransferase (α1,3/4-FucT) (Supplementary Fig. S1 online)^17^. Expression of only *FUT3* results in Le^a^ antigen expression, whilst expression of both *FUT3* and *FUT2* results in the expression of Le^b^ determinants on small intestinal glycans^17–19^.

To create a defined glycan model of the human small intestine to study the binding of ETEC CFA/I fimbriae to Lewis antigens, we glyco-engineered the well-defined and naturally HBGA devoid CHO-K1 cell line^22^. To generate CHO-K1 cells carrying Le^a^ or Le^b^ determinants on their cell surface, we expressed the extended core 1 glycan (GlcNAcβ3 Galβ3GalNAcα) enzyme B3GNT3, the type 1 chain glycan (Galβ 3GlcNAc)-encoding enzyme B3GALT5, and the Lewis gene-encoding enzyme FUT3 alone (generating Le^a^) or together with the H gene-encoding FUT1 (generating Le^b^) on a PSGL-1/mIgG2b fusion protein carrying probe (See Supplementary Fig. S1 and Table S1 online for further details)^20–24^. Expression of the B3GALT5 enzyme facilitates the biosynthesis of type 1 chains (Galβ3GlcNAc) on both *N*- and *O*-glycans (on the latter following co-expression of the extended core 1 enzyme B3GNT3), which is the obligate precursor for the Lewis (FUT3) and H-gene (FUT1) enzymes. This enabled us to engineer recombinant CHO-K1 cell lines carrying abundant Le^a^ or Le^b^ antigen substitutions^24^.

Initially, we evaluated the binding specificity of ETEC CFA/I fimbriae to our stable Lewis antigen-expressing cell lines, by infecting them with recombinant bacteria expressing CFA/I fimbriae. We found the CFA/I expressing strain attached more to the CHO-Le^a^ cell line, than to the CHO-Le^b^ or the Lewis antigen negative control cell lines (CHO-CP55 and CHO-K1). This may suggest that the α1,2-linked fucose of α1,2-FucT which is used to create Le^b^ could be either blocking the CFA/I binding sites on the Le^a^ receptor, or making the binding sites less accessible for the CFA/I fimbriae and thus preventing ETEC CFA/I attachment.

To define which subunit of ETEC CFA/I fimbriae are responsible for the binding of CFA/I fimbriae to our CHO-Le^a^ cell line, we also infected our cell lines with a recombinant *E.coli* strain expressing CFA/I without the minor subunit (Top10-CFA/IΔ*E*). As expected^7^, the *CfaE* deleted strain adhered more to CHO-Le^a^ cells than to CHO-Le^b^, CHO-CP55 or CHO-K1 cells. Similarly, pre-incubation of the Top10-CFA/I recombinant strain with either anti-CfaB or an equal mix of anti-CfaB and CfaE antibodies, significantly reduced CFA/I binding to CHO-Le^a^ cells. However, the bacterial binding after the pre-incubation of Top10-CFA/I with only anti-CfaE compared to no antibody pre-incubation was reduced, but not statically significant. As mature CFA/I fimbriae contain >1,000 copies of CfaB and one or a few copies of CfaE^4^, we therefore conclude the major subunit CfaB is naturally the more dominant subunit for CFA/I binding to small intestinal Le^a^ glycans.

To help understand why the CfaB subunit of ETEC CFA/I fimbriae binds to Le^a^ but not Le^b^ glycans, we performed computational molecular docking analysis. Our selected CFA/I docking site encompassed the amino acids V^24^EKNITVTASVD^35^ of the highly conserved CfaB Ig-like groove, found in major subunits of ETEC CFA/I related CFs (Supplementary Figs S2 and S5 online) and class 1 pili of bacteria that can cause urinary and respiratory infections by binding to host glycolipids containing HBGAs^9,17^.

Supporting our binding studies using glycan-defined CHO-K1 cells, as well as previous clinical observations^6,7^, our *in-silico* defined CFA/I docking site bound with a relatively higher binding affinity and higher interaction preference to the Le^a^ than to Le^b^ glycan. Moreover, the ligand strain energy as well as the small size of Le^a^-5, might explain its relative distortion in the CFA/I binding site as compared to that of the Le^b^-6 glycan. We hypothesise this relative distortion, could be one reason why multiple amino acids (Asn^27^,Thr^29^ and Glu^25^) of the CfaB Ig-like fold region (Supplementary Figs S2 and S5 online), docked to the α1,4-linked fucose and/or several surrounding moieties of the Le^a^-5 glycan. We now plan to perform more computational simulations as well as further *in-vitro* analysis to assess these observations further.

To highlight the flexibility of our small intestinal glycan defined like model, we also infected our Lewis expressing CHO-K1 cell lines with wild-type ETEC reference strains expressing CFA/I as well as CFA/I related CFs; the latter having been suggested to also bind to the small intestinal mucosa of children expressing Le^a^^7^. As expected, the wild-type ETEC strain expressing CFA/I bound significantly more to CHO-Le^a^ cells than to the Le^b^ or negative cell lines. Similarly, the ETEC strains expressing the related CFs CS1, CS2, CS4, CS14, but not CS17, bound more to CHO-Le^a^ cells.

Interestingly, the amino acids that we hypothesise by molecular docking to be important for CfaB binding to Le^a^-5, are also highly conserved in the major subunits of the related CFA/I CFs we have studied (see Supplementary Fig. S6 online for more details). However, the binding capacity differences of these wild-type ETEC strains expressing these CFs to CHO-Le^a^ cells may be due to evolved conformational differences amongst family members (Supplementary Fig. S6 online)^4^. Therefore, one reason why the CS17 ETEC strain does not bind to CHO-Le^a^ cells is that amino acid Arg^31^, located only a few positions away from the polar Asn^27^ and Thr^29^ amino acids (R group, Supplementary Fig. S6 online), is potentially leading to CS17 CF structural and conformational changes near these predicted binding sites.

**Figure 6:**
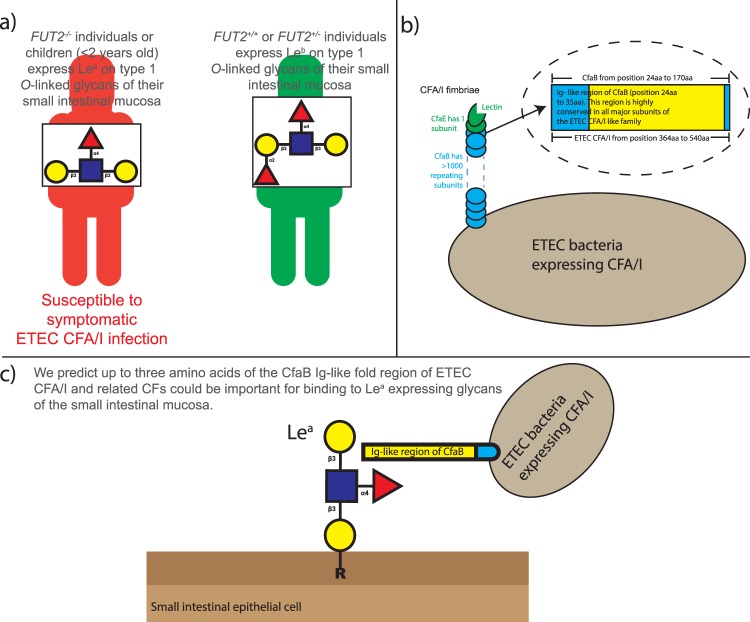
Working hypothesis. (**a**) *FUT2*^*−/−*^ individuals or children (<2 years old) express Le^a^ on type 1 *O*-linked glycans of the small intestinal mucosa. We have evidence to suggest they are susceptible to symptomatic ETEC CFA/I (and related CFA/I CF family members) infection. *FUT2*^+*/*+^ or *FUT2*^+*/−*^ individuals (>2 years old) express Le^b^ on type 1 *O-*linked glycans of the small intestinal mucosa. We have not found these individuals to not be susceptible to ETEC CFA/I (and related CFA/I CF family members) infection. (**b**) ETEC CFA/I fimbriae contains >1,000 copies of a major subunit (CfaB), with one or a few copies of the tip residing minor subunit (CfaE). A 12-aa stretch V^24^EKNITVTASVD^35^ of the Ig-like binding groove region of CfaB served as a CFA/I docking site our *in-silico* docking analysis. This 12 amino acid stretch also shares structural similarities with all ETEC CF major subunits of the CFA/I CF like family, and with class 1 pili from bacteria that can cause urinary and respiratory infections by binding to host glycolipids containing HBGAs. (**c**) Using our small intestinal like glycan defined CHO-K1 cell lines, we have demonstrated that CfaB of ETEC CFA/I fimbriae, as well as four related CFs, bind more to our CHO-K1 cell-line expressing Le^a^, compared to cells carrying Le^b^ or the CHO-K1 wild-type glycan phenotype. Using *in-silico* docking analysis, we predict up to three amino acids (Glu^25^, Asn^27^, Thr^29^) found in the immunoglobulin (Ig)-like groove region of CfaB of CFA/I and related CF fimbriae, could be important for the preferential and higher affinity binding of CFA/I fimbriae to Le^a^ glycans. These findings may lead to a better molecular understanding of ETEC pathogenesis, aiding in the development of vaccines and/or anti-infection therapeutics, which block such host-pathogen interactions.

A critical first step in a microbial pathogenesis is frequently the attachment to host cell glycans. In particular, targets on host tissues include the ubiquitously expressed HBGAs of different mucosae^17–19,28^. Structural and functional studies are now starting to reveal insights into why individuals expressing different HBGAs are at an increased risk of infections such as those caused by *Vibrio cholera*, *Pseudomonas aeruginosa*, norovirus, rotavirus and *Helicobacter pylori*^17,28^. By developing Lewis antigen cell-based models of the human small intestine, as well as performing docking studies, we have further defined why HBGA Le^a^ – expressing non-secretor (*FUT2*^−*/*−^) individuals, or young children (<2 years of age) are more susceptible to ETEC expressing CFA/I, or other related CFs family members^5,6,8,28^. Subsequently, our understanding and characterisation of these host-pathogen binding patterns could represent a critical point at which the adherence of ETEC expressing CFA/I and related CF family members, can be prevented with vaccines and/or anti-infection therapeutics that block this interaction (Fig. 6).

## Materials and Methods

### Plasmids used to construct glyco-engineered cell lines

Plasmids encoding the P-selectin glycoprotein-1/mouse immunoglobulin IgG2b Fc fragment (PSGL-1/mIgG2b) fusion protein, the human extended core 1 (GlcNAcβ3Galβ3GalNAcα) enzyme (B3GNT3), the type 1 chain (Galβ3GlcNAc) enzyme (B3GALT5), the Lewis gene α3/4-fucosyltransferase (FUT3) and the blood group H gene-encoded α2-fucosyltransferase (FUT1) were constructed as described previously (See Supplementary Table S1 online for details of the constructed plasmids)^20–22,24^. The pCMV/FUT1/Zeo rather than a FUT2 expression plasmid was selected to be used in this study, as it has been shown to better support Le^b^ expression in CHO cells^24^.

### Construction of glyco-engineered cell lines

Adherent CHO-K1 cells (ATCC^®^, Manassas, VA, USA) were seeded in six-well cell culture plates containing Dulbecco’s Modified Eagle’s medium (DMEM, Lonza), supplemented with 10% fetal bovine serum (FBS, Invitrogen, Waltham, MA, USA). All transfection experiments were performed 24 hours after seeding (70–80% cellular confluency). Cellular transfection was performed in accordance with manufacturer’s instructions using Lipofectamine 2000 (Invitrogen). CHO-K1 cells were transfected with the pEF1α/PSGL-1/mIgG2b/PAC plasmid to create the CHO-CP55 cell line. CHO-CP55 cells were then co-transfected with a cocktail of plasmids; pCMV/C1-β1,3GlcNAcT/Neo (human extended core 1 enzyme), pCMV/GalT5/Gpt (type 1 chain enzyme), in combination with of pCMV/FUT3/Hyg for Le^a^ expression (CHO- Le^a^ cell line), and combined with pCMV/FUT1/Zeo for Le^b^ expression (CHO-Le^b^ cell line) (Supplementary Fig. S1 and Table S1 online). To generate stable transfectants, all the five expression vectors were linearized with *Avr* II (New England BioLabs, Ipswich, MA, USA), and equal concentrations of plasmids encoding glycosyltransferases were used.

Following transfection (48 hours), all the transfected cell lines were incubated in selection medium as stated in the Supplementary Materials and Methods online. Two weeks after transfection, drug resistant clones were picked and transferred to 96-well plates containing their corresponding selection medium and propagated. Le^a^ and Le^b^ expression was assessed by immunocytochemistry and Western blot using monoclonal antibodies specific for the Le^a^ and Le^b^ determinants (see Supplementary Materials and Methods online for further details). Two clones- CHO-PSGL1-le^a^-a1 and CHO-PSGL1-le^b^-b4 (Fig. 1) were selected, expanded and used for ETEC cell binding experiments.

### Bacterial strains used in infection experiments

The previously constructed recombinant Top10-CFA/I (Amp^R^) and Top10-CFA/I/E^−^ (Cm^R^) strains were cultured as previously described^7^. The following wild-type ETEC strains and natural mutants were also used: E3006 (258909-3; CFA/I^+^ ST^+^/LT^+^), E120 (60R936; CS1^+^, ST^−^/LT^−^), E3017 (58R957; CS2^+^, ST^−^/LT^−^), E3037 (62R486; CS4^+^, ST^−^/LT^−^), E3013 (CS14^+^, STh^+^/LT^−^), and E3014 (CS17^+^, ST^−^/LT^+^)^4,30^. All ETEC strains were cultured as previously described^7,30^. Expression of CFs was tested by agglutination assays using monoclonal CF antibodies^4,30^.

In preparation for infection experiments, bacterial cultures were centrifuged at 10,000 × g for 10 minutes. The bacterial pellets were then washed and re-suspended in PBS before being re-centrifuged. Recombinant strains were re-suspended in PBS and ETEC strains were re-suspended in 0.5% D-mannose/PBS solution at an OD 0.8/mL (680 nm) to block possible binding by Type I fimbriae.

### Infection of CHO cell lines

Each cell line was seeded at a concentration of 0.5 × 10^5^/mL into microscope well slides (open µ-Slide, Ibidi). Three days after seeding, the well culture media was removed, washed with warm PBS and then replaced with 275 µL of DMEM with 1% FBS and 2nM L-glutamine in preparation for infection experiments. Cell lines were infected in duplicate with 25 µL of the corresponding bacterial suspensions (∼25 MOI) for three hours in a humidified incubator at 37 °C and 5% CO_2_. Following three washes with PBS, cells were fixed in paraformaldehyde for 10 minutes before being washed a further two times with PBS. Fixed cells were stained by immunofluorescence (see below) to determine the number of adherent bacteria to the cell lines.

### Inhibition of binding of the Top10-CFA/I ETEC strain to engineered CHO cells using monoclonal antibodies

The monoclonal anti-CfaB and CfaE antibodies were a kind gift from Professor Weiping Zhang of Kansas State University, USA. The CHO-Le^a^ cell line and the recombinant Top10-CFA/I strain were prepared as described above. A suspension (0.8OD/mL) of the Top10-CFA/I strain was mixed with either a 1:50 dilution of (i) anti-CfaB, (ii) anti-CfaE, (iii) a mixture of anti CfaB and anti-CfaE or (iv) no antibody mix. The bacterial/antibody mixtures were then mixed thoroughly and incubated for 30 minutes at room temperature. Cell lines were then infected with the bacterial mixtures as described above.

### Immunoflorescence staining of infected cell lines and quantifiable immunofluorescence analysis

All microscopy was performed using the inverted LSM700 confocal microscope with Zeiss Zen Blue software. Infected cells were stained for immunofluorescence microscopy using the protocol stated in the Supplementary Materials and Methods online.

To quantify bacterial adhesion, bacteria were visualised using the Alexa Fluor 488 channel and the CHO cells with the DAPI channel. All immunofluorescence settings remained the same for each experiment performed. Using the Multiple Single Positions (Tiles) position array tool available in the Zen Blue microscope software, six random tiles of each microscopic slide chamber were taken at x20 magnification. For each experiment performed, a total of two chambers per CHO cell line and infection were imaged (i.e. 12 random tile scans per cell line and bacterial infection). Confocal images in Zen format were analysed using the Velocity 3D image software (PerkinElmer, California, USA). Bacterial adhesion was quantified by measuring the mean surface area of bacteria (Alexa Flour 488 channel fluorescence, µM^2^) and the mean surface area of CHO cells (DAPI channel fluorescence, µM^2^). Binding of bacteria to CHO cell lines was expressed as a percentage of the total mean surface area of bacteria divided by the total mean surface area of CHO cells multiplied by 100.

### Molecular docking

*In-silico* docking studies were performed using the Glide module^31^ version 6.2, in Schrödinger, LLC, New York, NY, 2017. The X-ray crystal structure of the fused complex containing the CfaE and CfaB subunits of ETEC CFA/I fimbriae at 2.3 Å was obtained from the RSCB Protein Data Bank (PDB ID: 3F83)^9,25,32^, and prepared for molecular docking using the Protein Preparation Wizard^33^ of the Maestro, version 10.5 of the Schrödinger, LLC, New York, NY, 2017 software. Le^a^-5 (CID:11051152) and Le^b^-6 (CID:91852492) structures were downloaded from PubChem^26,27^, and converted to 3D before being processed for *in-silico* experiments using the Ligprep module^34^ of the Schrödinger, Maestro v10.5 software. This module generated a number of conformers (32 conformers) for each structure based on various tautomers, stereochemistries, ionization states and checking various ring conformations at a pH range set between 7 ± 2, followed by energy minimization with OPLS-2005 force field^31^.

Molecular Docking was performed using the Receptor Grid Generation panel module of the Glide, Schrödinger, LLC, New York, NY, 2017 software. A 12-aa stretch V^24^EKNITVTASVD^35^ of the Ig-like binding groove region of CfaB was used to a build grid and served as our CFA/I docking site^9^. The extra precision (XP) method of Glide dock was used for the docking experiments of Le^a^-5 and Le^b^-6 conformers to the CFA/I docking site with the sampling of the ligand being kept flexible during docking^35^.

The relative binding affinity score of our CFA/I docking complex to Le^a^-5 and Le^b^-6 was calculated (based on the XP docked complexes) using the MM-GBSA method (Molecular mechanics with generalized born surface area), available in the Schrödinger’s tool Prime software (Schrödinger, LLC, New York, NY, 2017)^36,37^. Apart from the MM-GBSA (ΔG) energy, a few other parameters were studied to further elucidate the docking results. These include: Effective Binding Energy (see calculations (1) and (2) below) which rescales the binding affinity of the Ligand strain energy to represent the extent of ligand distortion, ligand solvation energy (Lig solv GB) to represent the binding energy of ligand in solvent, and Linear Interaction Energy to represent the extent of binding affinity of ligand towards protein over solvent (see calculation (3) below).

1. Effective Binding Energy I = MM-GBSA/molecular weight of ligand
  Molecular weight of Le^a^-5 ligand = 853.774
  Molecular weight of Le^b^-6 ligand = 999.916
2. Effective Binding Energy II = MM-GBSA/number of heavy atoms in ligand
  Number of heavy atoms in Le^a^-5 ligand = 58
  Number of heavy atoms in Le^a^-5 ligand = 68
3. Linear Interaction Energy = MM-GBSA − Ligand solvent GB.

Interesting docking sites were selected based on Effective Binding Energy (I and II), the number of interactions from the conserved region of CfaB, and docked complexes interacting with the Lewis antigen or neighbouring moieties of either Le^a^-5 or Le^b^-6.

### Statistical analysis

Binding of bacteria to the different CHO cell lines were expressed as mean percentages of added bacteria binding to the cells in 12 confocal tile scans. Inhibition results were obtained from two independent experiments performed in duplicate. All other binding experiments were obtained from at least three independent experiments performed in duplicate. Statistical analysis was performed using Graph Pad Prism 6. ANOVA using Dunn’s multi comparisons test was used to compare the mean percentage of bacteria binding to the CHO-Le^a^, CHO-Le^b^, CHO-CP55 and CHO-K1 cell lines. For additional verification, the Wilcoxon matched pairs sign rank test was used to compare the mean percentage of bacteria binding to the CHO-Le^a^ cell line compared to CHO-Le^b^ cell line. Significance was set at a P value of <0.05.

### Data availability

The raw datasets generated during and/or analysed during the current study are available in the Figshare repository, (https://figshare.com/s/41a7b658f9474b6dfd53).

## Electronic supplementary material

Supplementary Information
