# Supplementary material for: Glyco-engineered cell line and computational docking studies reveals enterotoxigenic Escherichia coli CFA/I fimbriae bind to Lewis a glycans
Source: Sci Rep. 2018 Jul 26;8:11250. doi: 10.1038/s41598-018-29258-0 (PMC6062558; doi:10.1038/s41598-018-29258-0)
Supplement: Supplementary file 1 — Supplementary Information [file 41598_2018_29258_MOESM1_ESM.pdf]

Supplementary Information

Glyco-engineered cell line and computational docking studies reveals enterotoxigenic *Escherichia coli* CFA/I fimbriae bind to Lewis a glycans

Lynda Mottram[1+*], Jining Liu[2+], Sonali Chavan[3], Joshua Tobias[1,4], Ann-Mari Svennerholm[1], Jan Holgersson[2]

[1]Department of Microbiology and Immunology at the Institute of Biomedicine, Sahlgrenska Academy, University of Gothenburg, Gothenburg, Sweden
[2]Department of Clinical Chemistry and Transfusion Medicine at the Institute of Biomedicine, Sahlgrenska Academy, University of Gothenburg, Gothenburg, Sweden
[3]Department of Chemistry and Molecular Biology, University of Gothenburg, Gothenburg, Sweden
[4]Institute of Specific Prophylaxis and Tropical Medicine, Medical University of Vienna, Vienna, Austria

[*] Corresponding author email address: lynda.mottram@gu.se
[+] These authors contributed equally to this work.

Supplementary Materials and Methods

The frequency of Le^a and Le^b expressing CHO cells was assessed by immunocytochemistry. The constructed CHO-Le^a and CHO-Le^b cell lines were split into six-well plates and cultured for two days. Cells were fixed in 30 % ice-cold acetone in methanol (v/v) for two minutes at room temperature, washed twice in PBS, and blocked in 1% BSA/PBS for 30 minutes. For detection of Le^a, cells were incubated with the anti-Le^a antibody (BG-5, mouse IgG; BioLegend) followed by three PBS washes and then an incubation with secondary Alexa Fluor 488-conjugated goat anti-mouse IgG (Jackson). For Le^b detection, cells were incubated with the anti-Le^b antibody (BG-6, mouse IgM; BioLegend), followed by three PBS washes and then an incubation with the secondary FITC-conjugated goat anti-mouse IgM (Jackson). Both primary antibodies were diluted 1:50 in 1% BSA/PBS and incubated for 1 hour at room temperature. The secondary antibodies were diluted 1:400 in 1% BSA/PBS and were incubated for 30 minutes in the dark. As a counterstain, cells were incubated for 1-2 minutes in DAPI (Invitrogen) at a concentration of 300 nM, before rinsing in PBS. Subsequently, the cells were analyzed in a fluorescence microscope (Olympus IX2; Olympus).

SDS-PAGE and Western blotting methods were used to verify the presence of Le^a and Le^b determinants on the P-selectin glycoprotein ligand-1/mouse IgG2b fusion proteins of the CHO-Le^a, CHO-Le^b and CHO-CP55 cells. Secreted recombinant PSGL-1/mIgG2b protein from 5 ml of medium was adsorbed onto 25µl goat anti-mouse IgG agarose beads (Sigma) at 4˚C overnight. After washing with PBS, beads with the bound fusion protein were re-suspended in 30µl LDS non-reducing sample buffer (Invitrogen AB) and denatured at 70˚C for 10 min. Then, 15µl of each sample was separated on 3-8% NuPAGE gels (Invitrogen) under non-reducing conditions. Separated proteins were blotted onto nitrocellulose membranes (Invitrogen) using the iBlot apparatus (Invitrogen). The membranes were blocked with PBS containing 0.2% Tween-20 (PBS-T) and 3% BSA, which was also used for the dilution of antibodies. For detection of PSGL-1, the membrane was incubated for one hour in a 1: 2,000 of mouse anti-CD162 antibodies (BD PharMingen) recognizing the N-terminal of PSGL-1. For detection of Le^a and Le^b, the anti-Le^a and anti-Le^b antibodies were diluted 1:500. Peroxidase-conjugated goat anti-mouse IgG (Jackson, West Grove, PA, USA) at a dilution of 1:10000 was used as secondary antibody and incubated at room temperature for one hour. Following each incubation, membranes were washed three times with PBS-T. Bound antibody was visualized by chemiluminescence using the ECL kit according to the manufacturer's instructions (Advansta, Menlo Park, California, USA).

Cell culture of CHO-K1 cells and glyco-engineered CHO-K1 cell lines. CHO-K1 was maintained and cultured in DMEM supplemented with 10% FBS, 2mM L-glutamine (Invitrogen) and 10mL/L Penicillin/Streptomycin Solution (Sigma). The CHO-CP55 cell line (S1 Fig. and S1 Table) was maintained in the same culture medium together with 3µg/mL puromycin. The CHO-Le^a and CHO-Le^b cell lines (see Supplementary Fig 1 and Supplementary Table 1), were maintained in the same culture medium as the CHO-CP55 cell line, together with 600µg/mL G418, 200µg/mL hygromycinB, 25µg/mL mycophenolic acid, 0.25mg/mL xanthine, and 13.6µg/mL hypoxanthine. The CHO-Le^b cell line was maintained in the same culture medium as the CHO-Le^a cell line, but with an addition of 50µg/mL zeocin. Culture media was replaced every two to three days. Cells were maintained in a humidified

incubator at 37°C and 5% CO_2. The frequency of cell line Le^a and Le^b expression was checked monthly using the supplementary method described above in the "frequency of Le^a and Le^b expressing CHO cells assessment by immunocytochemistry" section.

Immunofluorescence staining protocol for infected CHO-Le^a, CHO-Le^b, CHO-CP55 and CHO-K1 cells. Fixed cells were blocked in 1% BSA/PBS for 30 minutes. For detection of bacteria, either the mouse anti CFA/I[24] (concentration 1:50), or the goat anti *E.coli* (Abcam, Cambridge, UK; concentration 1:200) antibodies mixed in blocking buffer were used. Following a one-hour incubation and three PBS washes, either the secondary Alexa Fluor 488-conjugated goat anti-mouse IgG (Jackson) or the secondary Alexa Fluor 488-conjugated rabbit anti-goat IgG (Abcam) were added for detection of bound primary antibodies. The cells were then washed another three times in PBS. For detection of Le^a or Le^b, the anti-Le^a and Le^b antibodies were applied as previously described and then washed three times in PBS. Cells stained with the Le^a antibody were incubated with a secondary biotinylated goat anti-mouse IgG (Southern Biotech, Birmingham, AL, USA; concentration 1:200), followed by another three PBS washes, and a 30-minute incubation with Alexa Fluor 647-conjugated streptavidin (Life technologies; concentration 1:500), followed by a further three PBS washes. Cells stained with the Le^b antibody were incubated with a secondary Texas Red-conjugated goat anti-mouse IgM (Southern Biotech; concentration 1:200), followed by three PBS washes. As a counterstain the Prolong diamond Antifade Mountant with DAPI (Thermofisher, Waltham, MA, USA) nucleic acid stain was used. Cells were analyzed in a confocal microscope (inverted LSM700, Carl Zeis).

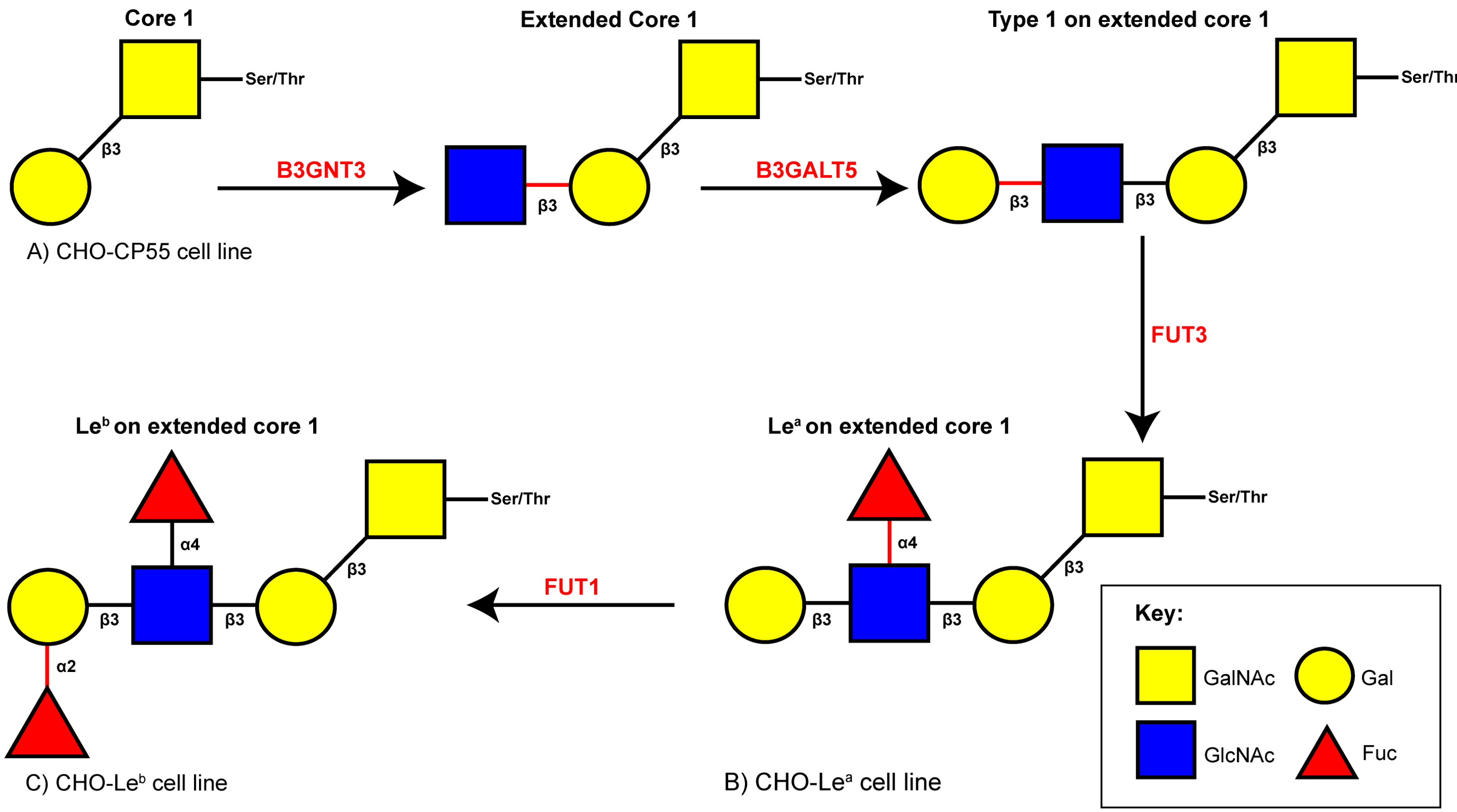

Supplementary Fig. S1. The biosynthetic pathway of Lewis antigens generated in the glyco-engineered CHO-K1 cell lines. CHO-K1 cells were transfected with the PGSL-1/mIgG2b expression plasmid to create the A) CHO-CP55 cell line. CHO-CP55 cells were then co-transfected with plasmids encoding the B3GNT3, B3GALT5 and FUT3 enzymes without or with FUT1 to generate the extended core 1 (GlcNAcb3Galb3GalNAca) chain, extended with a type 1 chain (Galb3GlcNAc) and terminated with Le^a [B) CHO-Le^a cells] or Le^b [C) CHO-Le^b cells] determinants, respectively.

Expression plasmid	cDNA	Resistance gene	Source
pEF1α/PSGL-1/mIgG2b/PAC[a]	PSGL-1/mIgG2b fusion gene	Puromycin acetyl transferase (puromycin resistance)	HL-60 cDNA library
pCMV/C1-β1,3GlcNAcT/Neo[b]	B3GNT3	pSV2neo (G418 resistance)	HT-29 cDNA library
pCMV/GalT5/Gpt[c]	B3GALT5	Guanosine phosphoribosyl transferase (mycophenolic acid, xanthine and hypoxanthine resistance)	Human placental genomic DNA
pCMV/FUT3/Hyg[d]	FUT3	Hygromycin B phosphotransferase (hygromycin B resistance)	HT-29 cDNA library
pCMV/FUT1/Zeo[d]	FUT1	Streptoalloteichus hindustanus bleomycin (zeocin resistance)	Human placental genomic DNA

[a] Construction described in: Liu J, Qian Y, Holgersson J. Removal of xenoreactive human anti-pig antibodies by absorption on recombinant mucin-containing glycoproteins carrying the Gal alpha1,3Gal epitope. Transplantation. 1997;63(11):1673-82.

[b] Construction described in: Liu J, Holgersson J. Recombinant Galalpha1,3Gal-substituted mucin/immunoglobulin chimeras: a superior absorber of anti-pig antibodies. Transplant Proc. 2000;32(5):859.

[c] Construction described in: Liu J, Jin C, Cherian RM, Karlsson NG, Holgersson J. O-glycan repertoires on a mucin-type reporter protein expressed in CHO cell pools transiently transfected with O-glycan core enzyme cDNAs. J Biotechnol. 2015;199:77-89.

[d] Construction described in: Holgersson J, Lofling J. Glycosyltransferases involved in type 1 chain and Lewis antigen biosynthesis exhibit glycan and core chain specificity. Glycobiology. 2006;16(7):584-93

Supplementary Table S1. Expression plasmids used to glyco-engineer CHO-K1 cells.

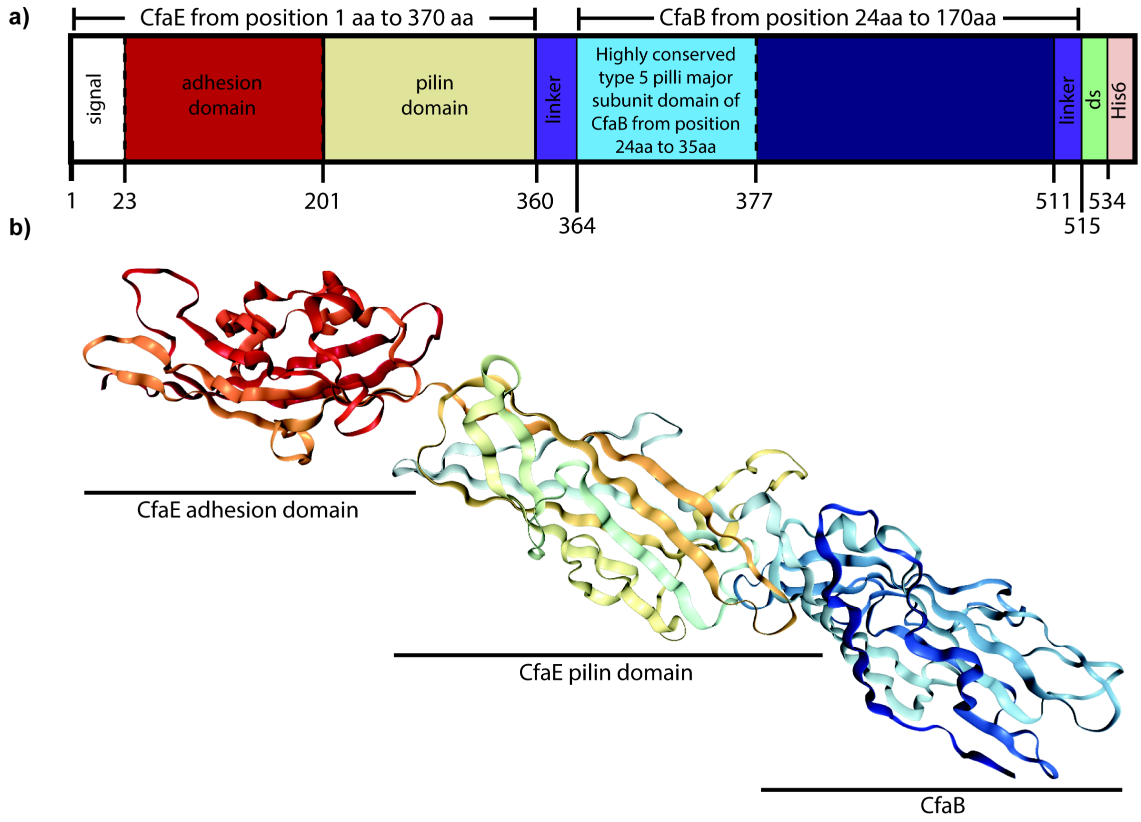

Supplementary Fig. S2. Cartoon depictions of the ETEC CFA/I recombinant fusion protein used for molecular docking analysis. a) Schematic depiction of the recombinant CfaE and CfaB fusion protein. Position 364 to 377 (CfaB position 24 to 35) was used to build a grid and serve as a docking site our molecular docking analysis. b) 3D cartoon representation of the structure of CFA/I showing the minor subunit CfaE and the major subunit CfaB. The 3D structure of CFA/I was downloaded from the RCSB Protein databank and used for molecular docking experiments (accession number: 3F83). Further details of how the atomic structure of ETEC CFA/I was determined can be found in the following publication. Li Y, Poole S, Nishio K, Jang K *et al*,. Structure of CFA/I fimbriae from enterotoxigenic *Escherichia coli*. PNAS. 2009; 106(26):10793-10798.

Pose I.D	XP Glide Score	MM-GBSA Score	Ligand Solvent GB	Ligand Strain energy	Effective Binding Energy I[a]	Effective Binding Energy II[b]	Linear Interaction Energy[c]
a01	-8.766	-53.727	-34.405	11.242	-0.063	-0.926	-19.322
a02	-9.174	-52.904	-40.411	6.556	-0.062	-0.912	-12.493
a03	-8.898	-52.583	-32.956	7.368	-0.062	-0.907	-19.627
a04	-10.036	-52.168	-40.076	15.161	-0.061	-0.899	-12.092
a05	-9.993	-51.726	-32.932	1.337	-0.061	-0.892	-18.794
a06	-10.182	-49.319	-37.542	18.196	-0.058	-0.850	-11.777
a07	-9.316	-49.141	-28.243	3.686	-0.058	-0.847	-20.897
a08	-10.310	-45.768	-32.258	14.674	-0.054	-0.789	-13.509
a09	-8.005	-45.628	-37.112	10.394	-0.053	-0.787	-8.517
a10	-8.303	-44.806	-34.666	14.544	-0.052	-0.773	-10.139
a11	-9.761	-43.262	-30.705	11.525	-0.051	-0.746	-12.557
a12	-9.450	-42.867	-39.505	10.614	-0.050	-0.739	-3.362
a13	-9.300	-42.491	-32.738	14.453	-0.050	-0.733	-9.753
a14	-9.177	-41.595	-29.892	25.161	-0.049	-0.717	-11.703
a15	-8.275	-39.682	-36.887	13.194	-0.046	-0.684	-2.797
a16	-9.230	-39.556	-34.781	14.609	-0.046	-0.682	-4.776
a17	-8.246	-38.663	-31.552	20.441	-0.045	-0.667	-7.111
a18	-7.369	-38.423	-29.190	14.308	-0.045	-0.662	-9.233
a19	-8.556	-37.949	-29.054	14.339	-0.044	-0.654	-8.893
a20	-7.716	-35.491	-31.047	17.212	-0.042	-0.612	-4.444
a21	-8.939	-34.209	-34.719	12.187	-0.040	-0.590	0.509
a22	-10.030	-33.741	-34.884	17.366	-0.040	-0.582	1.143
a23	-9.106	-33.256	-30.492	22.033	-0.039	-0.573	-2.766
a24	-9.851	-33.245	-32.461	28.363	-0.039	-0.573	-0.784
a25	-9.959	-31.349	-30.261	20.182	-0.037	-0.540	-1.089
a26	-8.853	-31.151	-34.541	25.857	-0.036	-0.537	3.390
a27	-8.661	-27.621	-27.188	22.216	-0.032	-0.476	-0.433
a28	-8.071	-27.076	-29.072	23.890	-0.032	-0.467	1.996
a29	-9.634	-24.820	-27.993	14.860	-0.029	-0.428	3.172
a30	-9.588	-24.467	-31.663	40.362	-0.029	-0.422	7.197
a31	-8.848	-21.628	-30.883	26.645	-0.025	-0.373	9.255
a32	-10.906	-17.683	-32.226	32.367	-0.020	-0.305	14.543

[a] = MM-GBSA score / molecular weight of glycan

[b] = MM-GBSA score / number of heavy atoms in glycan

[c] = MM-GBSA score - Ligand solvent GB

Supplementary Table S2. The 32 Le^a-5 docking poses identified by *in-silico* docking analysis. Results are ranked based on highest Effective Binding Energy I and II. The ten most likely (highlighted in yellow) candidate poses were selected for further analysis. All energy units above are measured in kcal/mol.

Pose I.D	XP Glide Score	MM-GBSA score	Ligand Solvent GB	Ligand Strain energy	Effective Binding Energy I[a]	Effective Binding Energy II[b]	Linear Interaction Energy[c]
b01	-8.302	-56.176	-53.394	0.935	-0.056	-0.826	-2.782
b02	-8.798	-55.204	-36.825	0.081	-0.055	-0.812	-18.379
b03	-9.030	-52.104	-44.538	17.504	-0.052	-0.766	-7.5661
b04	-10.087	-48.964	-38.530	10.353	-0.049	-0.720	-10.435
b05	-8.395	-48.488	-38.728	11.456	-0.048	-0.713	-9.759
b06	-8.489	-46.655	-39.143	10.470	-0.047	-0.686	-7.511
b07	-8.046	-46.198	-39.974	5.267	-0.046	-0.679	-6.224
b08	-9.352	-44.813	-41.044	5.922	-0.0452	-0.659	-3.769
b09	-7.834	-44.394	-37.250	8.935	-0.044	-0.653	-7.143
b10	-7.784	-44.001	-37.422	5.502	-0.044	-0.647	-6.579
b11	-6.856	-43.605	-45.680	-3.474	-0.044	-0.641	2.075
b12	-8.646	-42.455	-44.355	10.748	-0.042	-0.624	1.901
b13	-9.810	-41.492	-40.758	4.040	-0.042	-0.610	-0.734
b14	-8.861	-41.071	-40.278	20.515	-0.041	-0.604	-0.793
b15	-8.120	-40.569	-39.216	1.847	-0.041	-0.597	-1.353
b16	-10.467	-39.838	-40.587	8.593	-0.040	-0.586	0.749
b17	-9.386	-38.703	-32.152	2.815	-0.039	-0.569	-6.551
b18	-8.358	-38.663	-37.911	17.356	-0.039	-0.569	-0.752
b19	-10.538	-37.305	-40.086	16.509	-0.037	-0.549	2.782
b20	-9.230	-37.188	-32.846	15.566	-0.037	-0.547	-4.341
b21	-8.839	-35.000	-31.410	20.315	-0.035	-0.515	-3.590
b22	-9.351	-34.409	-42.397	2.691	-0.034	-0.506	7.988
b23	-7.555	-31.597	-42.760	2.279	-0.032	-0.465	11.163
b24	-10.838	-30.565	-40.001	30.925	-0.031	-0.449	9.436
b25	-8.745	-29.334	-33.864	22.649	-0.029	-0.431	4.530
b26	-9.206	-28.688	-23.734	15.319	-0.029	-0.422	-4.953
b27	-9.106	-27.321	-35.322	21.434	-0.027	-0.402	8.001
b28	-12.243	-25.058	-45.027	21.317	-0.025	-0.369	19.969
b29	-7.487	-25.011	-38.980	10.993	-0.025	-0.368	13.969
b30	-7.811	-24.670	-37.537	16.076	-0.025	-0.363	12.867
b31	-9.752	-21.226	-23.607	21.005	-0.021	-0.312	2.381
b32	-8.481	-19.870	-34.613	28.067	-0.020	-0.292	14.743

[a] = MM-GBSA score / molecular weight of glycan
[b] = MM-GBSA score / number of heavy atoms in glycan
[c] = MM-GBSA score - Ligand solvent GB

Supplementary Table S3. The 32 Le^{b}-6 docking poses identified by *in-silico* docking analysis. Results are ranked based on highest Effective Binding Energy I and II. The ten most likely (highlighted in yellow) candidate poses were selected for further analysis. All energy units above are measured in kcal/mol.

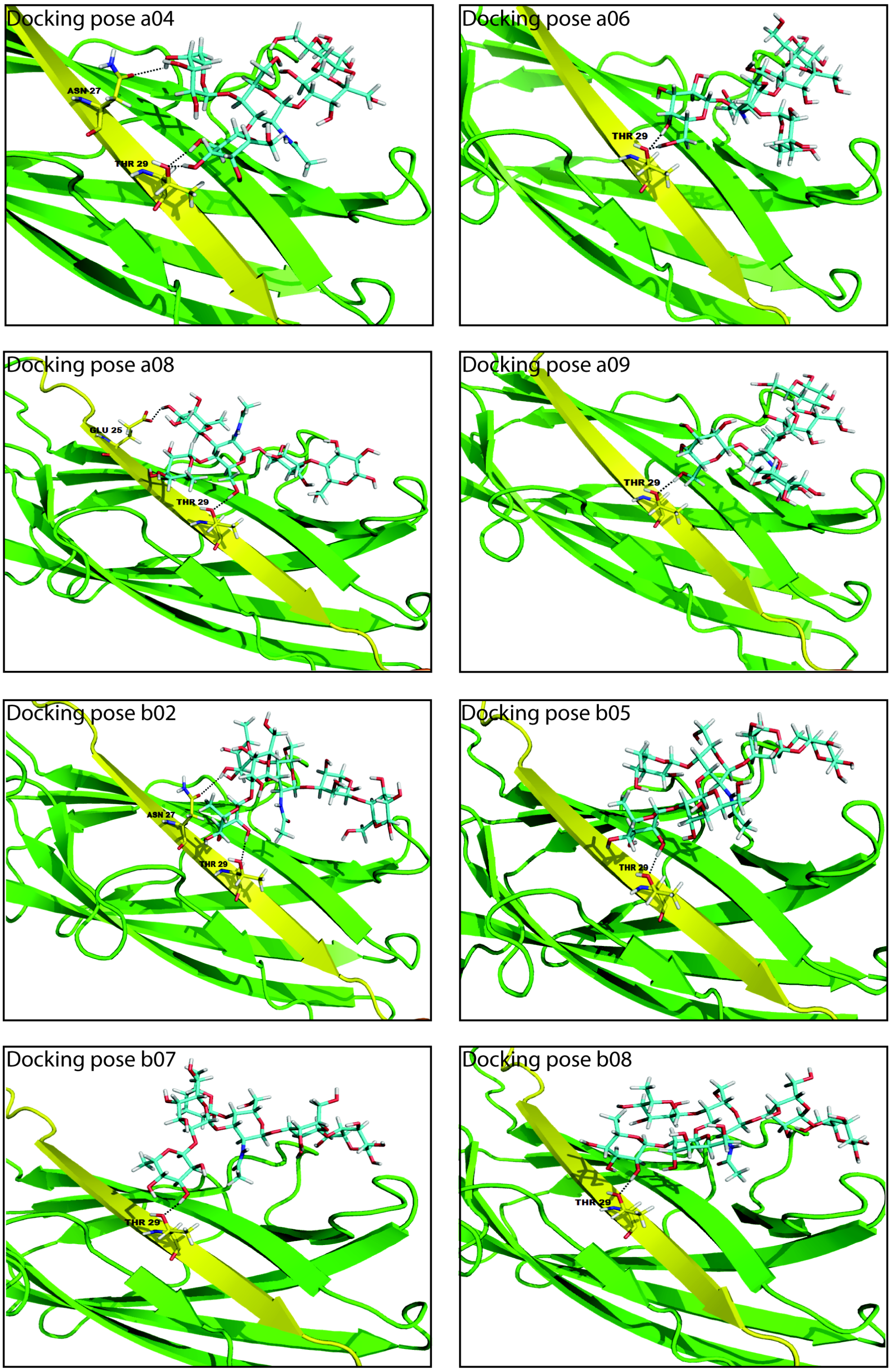

Supplementary Fig. S3. Ribbon diagrams of other Le^{a}-5 and Le^{b}-6 candidate docked poses, predicted to bind around the Le^{a} or Le^{b} moieties of the Le^{a}-5 and Le^{b}-6 ligands. Docking is with either Le^{a}-5 or Le^{b}-6 (Cyan stick) glycans, with hydrogen bond interactions (dotted line) to three different immunogenic CfaB epitopes; Glu^{25}, Asn^{27} and Thr^{29} (yellow stick). CfaE is the green ribbon and the N terminal domain containing the Ig-like groove of CfaB is the yellow ribbon.

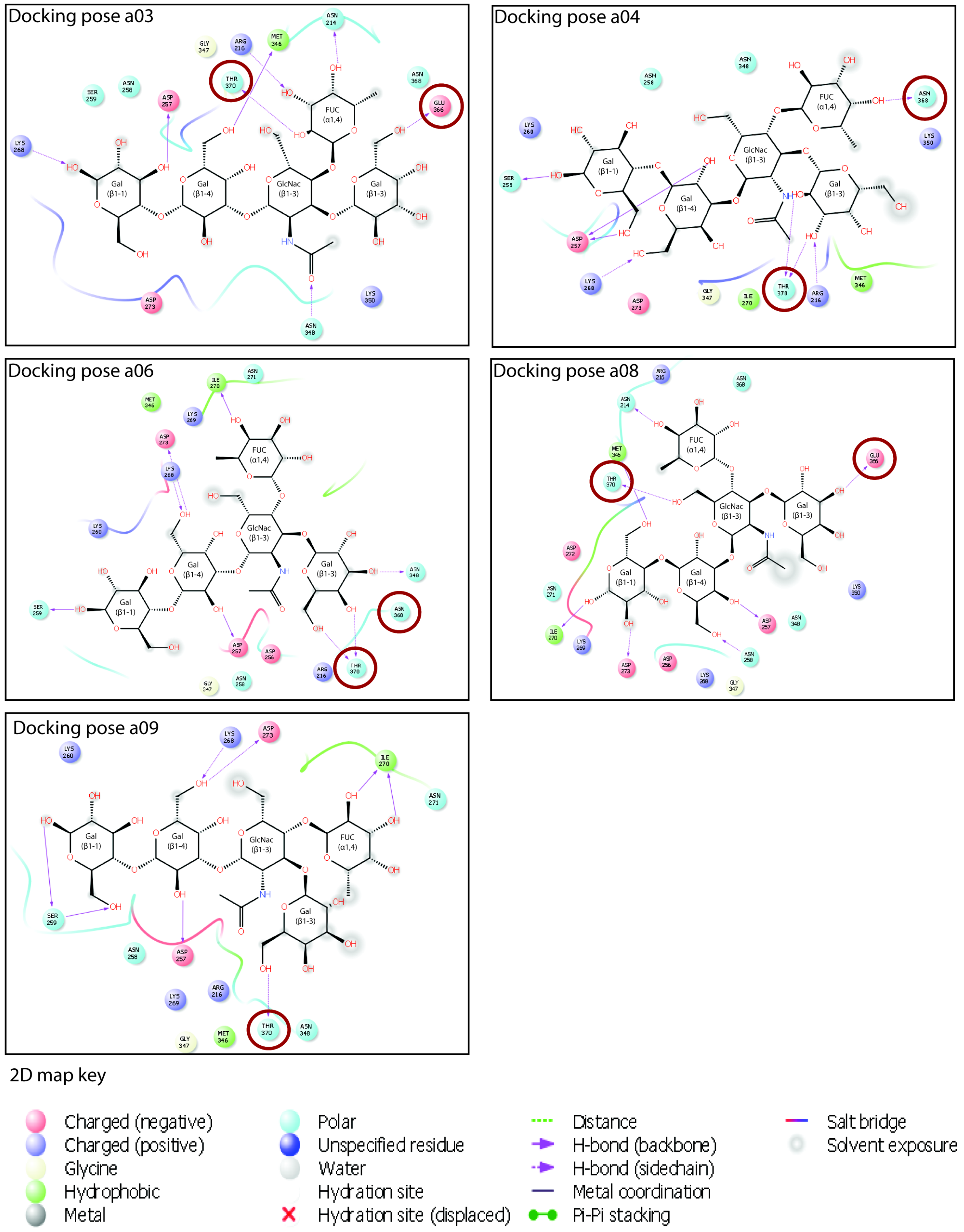

Signifies amino acid of interest from docking analysis with CFA/I fusion protein (PDB ID:3F83). THR370 = THR29, ASN368 = ASN27 and GLU366 = GLU25 on the CfaB major subunit of CFA/I fimbriae.

Supplementary Fig. S4. 2D docking diagrams of selected Le^a-5 candidate docked poses. Predicted CfaB binding to the α1,4Fuc and/or neighboring β1,3Gal and β1,3GalNAc moieties.

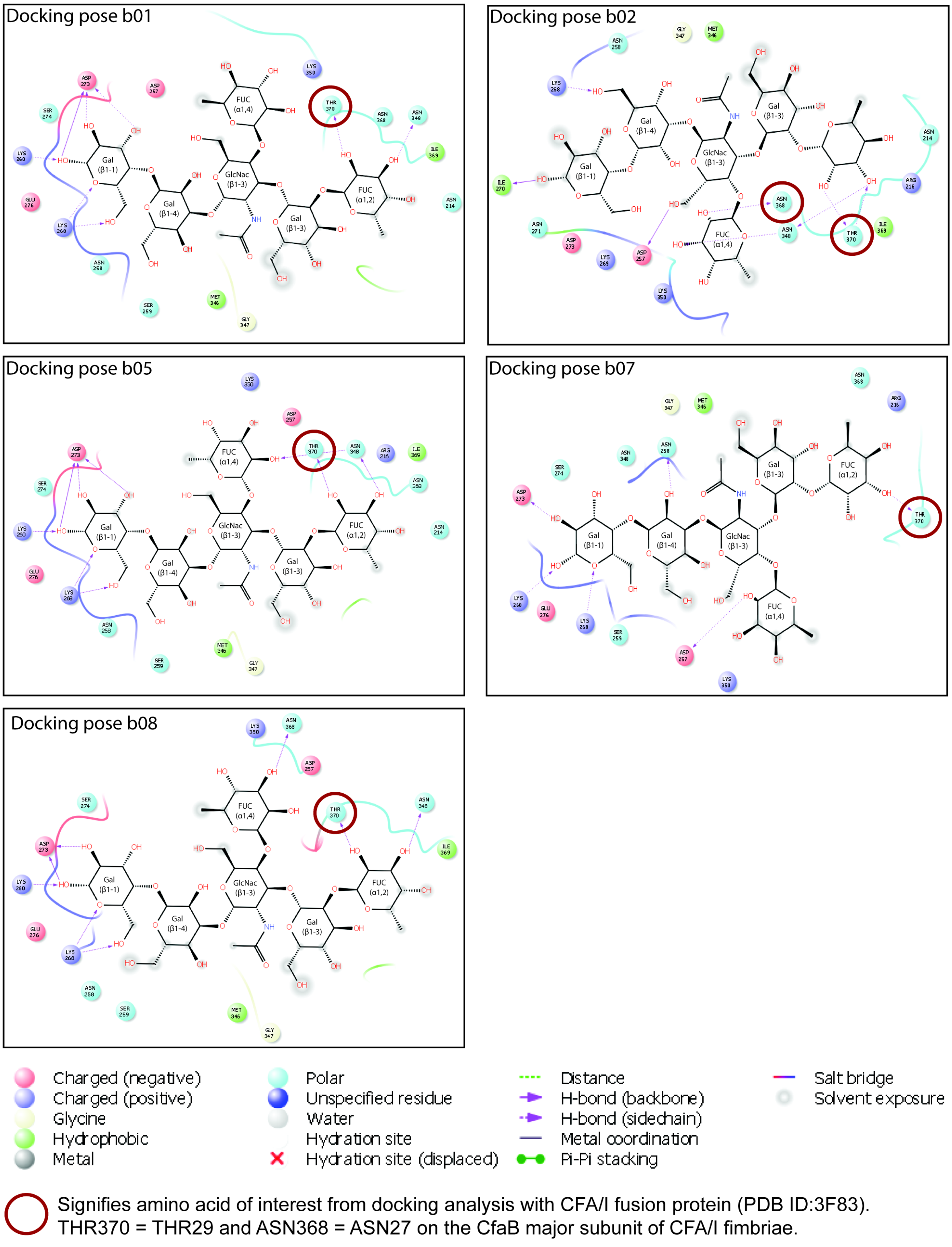

Supplementary Fig. S5. 2D docking diagrams of selected Le^{b}-6 candidate docked poses. Predicted CfaB binding to Lewis antigen moieties.

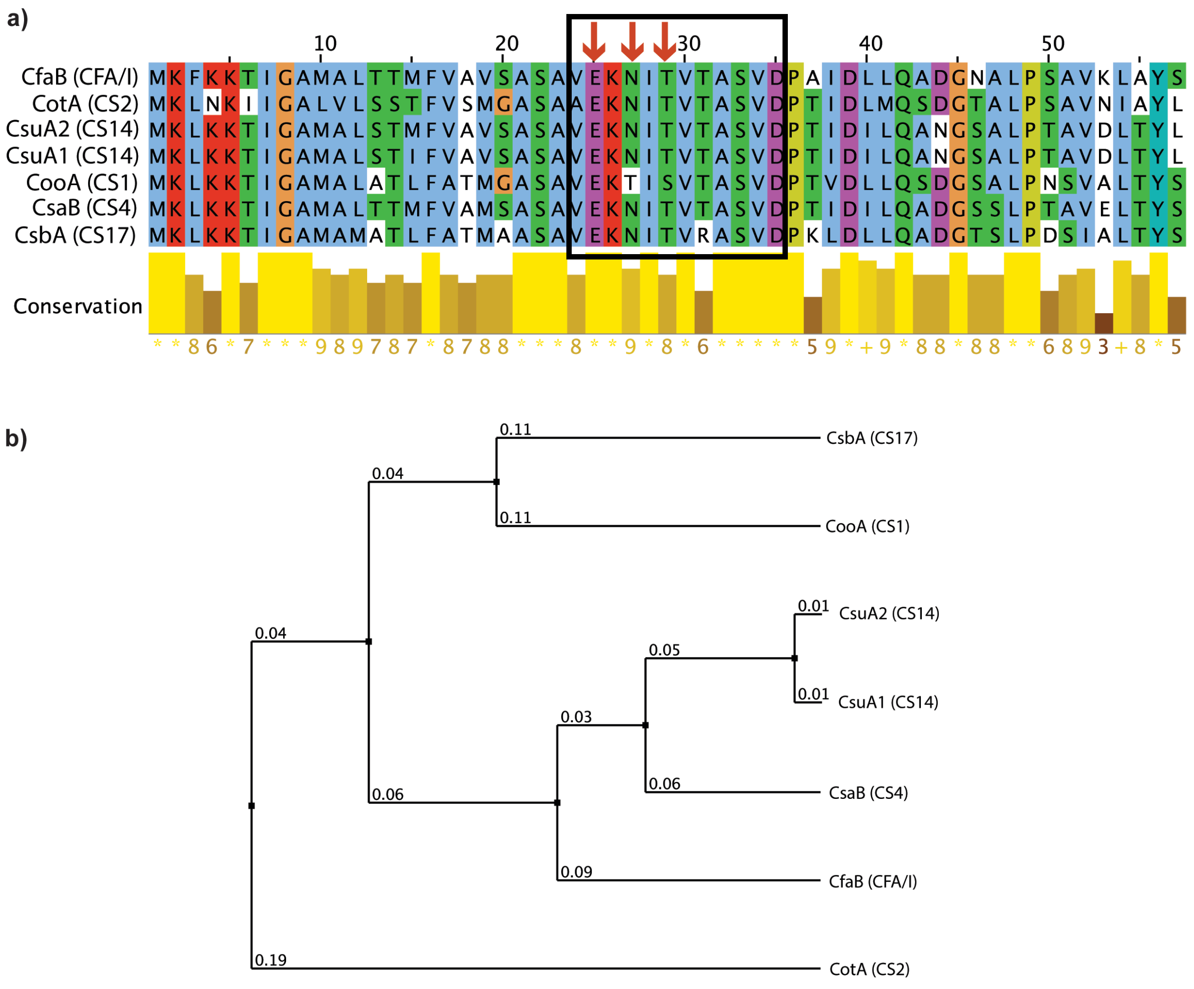

Supplementary Fig. S6. Multiple sequence protein alignments and alignment tree, of the N terminal domain of the major subunits of the ETEC CFs used in this study. a) Clustal X protein sequence alignment of the ETEC CF major subunits from 1aa-56aa. Amino acids are coloured as: Blue for Hydrophobic, Green for Polar, Orange for Glycine, Yellow for Proline and White for un-conserved. The Jalview conservation scores and colour schemes are assigned from 0 (white) to 10 (*, most intense yellow) based on the common physico-chemical properties of the residues. Red arrows indicate the conserved amino acids of the Ig-like groove of CfaB (Glu^{25}, Asn^{27} and Thr^{29}), that are predicted to bind to Le^{a}-5 during the *in silico* docking analysis. b) Clustal X protein alignment tree of the major subunits from 1aa-56aa, showing the evolutionary relationship distances of each of the CFs. The sequences were downloaded from UniProt: CfaB of CFA/I (P0CK93), CooA of CS1 (Q6JAY9), CsbA of CS17 (Q848J7), CsaB of CS4 (Q93G69), CotA of CS2 (Q47117), CsuA1 of CS14 (Q5SGE9) and CsuA2 of Cs14 (Q5SGE8). This analysis incorporates the highly conserved V^{24}EKNITVTASVD^{35} type 5 pili ETEC CF major subunit region (highlighted in black box in a)) that was originally described by Li Y.F *et al.*, Structure of CFA/I fimbriae from enterotoxigenic Escherichia coli. *Proc Natl Acad Sci USA* **106**, 10793-10798 (2009).

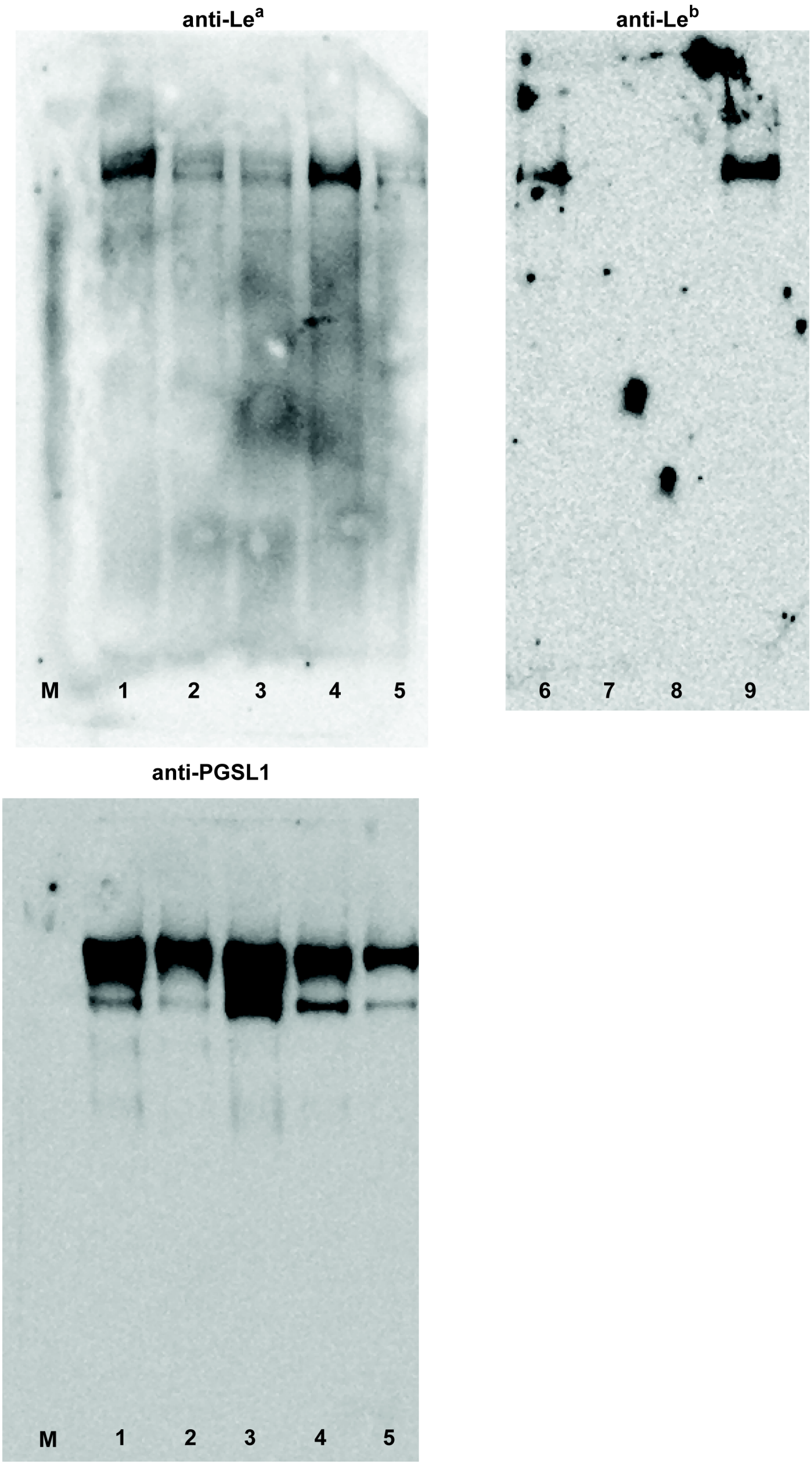

Fig. S7. Raw Western blot analysis images of PSGL-1/mIgG2b proteins expressed in the CHO-CP55, CHO-Le^a and CHO-Le^b cell lines.

SDS-PAGE and Western blot analysis of PSGL-1/mIgG2b proteins expressed in the Le^a positive control (lane 1), Le^b positive control (lanes 2 and 6) CHO-CP55 cell lines (lanes 3 and 7) CHO-Le^a cell lines (lanes 4 and 8) and CHO-Le^b cell lines (lanes 5 and 9). In each lane, 1.5µg of protein was loaded. Membranes were probed with either anti-PGSL1, anti-Le^a or anti Le^b antibodies followed by an anti-mouse IgG secondary antibody. M= protein ladder.
